# Brain texture alterations predict subtle visual perceptual dysfunctions in recent onset psychosis and clinical high-risk state

**DOI:** 10.1038/s41398-026-03840-x

**Published:** 2026-02-12

**Authors:** Rebekka Lencer, Andreas Sprenger, Inga Meyhöfer, Udo Dannlowski, Georg Romer, Lana Kambeitz-Ilankovic, Joseph Kambeitz, Theresa Lichtenstein, Marlene Rosen, Stephan Ruhrmann, Shalaila S. Haas, Raimo K. R. Salokangas, Christos Pantelis, Carolina Bonivento, Frauke Schultze-Lutter, Eva Meisenzahl, Paolo Brambilla, Alessandro Bertolino, Rachel Upthegrove, Christos Davatzikos, Nikolaos Koutsouleris, Stefan Borgwardt, Christina Andreou, Alexandra Korda, the PRONIA Consortium, Raimo K. R. Salokangas, Alessandro Bertolino, Nikolaos Koutsouleris, Anne Ruef, Lisa Hahn, Dominic B. Dwyer, Shalaila Haas, Linda A. Antonucci, Alkomiet Hasan, Claudius Hoff, Ifrah Khanyaree, Aylin Melo, Susanna Muckenhuber-Sternbauer, Yanis Köhler, Ömer Öztürk, Nora Penzel, David Popovic, Adrian Rangnick, Sebastian von Saldern, Rachele Sanfelici, Moritz Spangemacher, Ana Tupac, Maria Fernanda Urquijo-Castro, Johanna Weiske, Antonia Wosgien, Camilla Krämer, Lana Kambeitz-Ilankovic, Joseph Kambeitz, Julian Wenzel, Stephan Ruhrmann, Karsten Blume, Dennis Hedderich, Dominika Julkowski, Nathalie Kaiser, Thorsten Lichtenstein, Ruth Milz, Alexandra Nikolaides, Tanja Pilgram, Mauro Seves, Martina Wassen, Christina Andreou, Stefan Borgwardt, André Schmidt, Anita Riecher-Rössler, Laura Egloff, Fabienne Harrisberger, Ulrike Heitz, Claudia Lenz, Letizia Leanza, Amatya Mackintosh, Renata Smieskova, Erich Studerus, Anna Walter, Sonja Widmayer, Stephen J. Wood, Rachel Upthegrove, Paris Alexandros Lalousis, Chris Day, Sian Lowri Griffiths, Mariam Iqbal, Mirabel Pelton, Pavan Mallikarjun, Alexandra Stainton, Ashleigh Lin, Jarmo Hietala, Alexander Denissoff, Anu Ellilä, Tiina From, Markus Heinimaa, Tuula Ilonen, Päivi Jalo, Heikki Laurikainen, Antti Luutonen, Akseli Mäkela, Janina Paju, Henri Pesonen, Reetta-Liina Säilä, Anna Toivonen, Otto Turtonen, Frauke Schultze-Lutter, Eva Meisenzahl, Alexandra Korda, Susanne Neufang, Christian Schmidt-Kraepelin, Henrik Rohner, Sonja Botterweck, Norman Kluthausen, Gerald Antoch, Julian Caspers, Hans-Jörg Wittsack, Pierluigi Selvaggi, Giuseppe Blasi, Giulio Pergola, Grazia Caforio, Leonardo Fazio, Tiziana Quarto, Barbara Gelao, Raffaella Romano, Ileana Andriola, Andrea Falsetti, Marina Barone, Roberta Passiatore, Marina Sangiuliano, Rebekka Lencer, Marian Surmann, Olga Bienek, Udo Dannlowski, Ana Beatriz Solana, Manuela Abraham, Timo Schirmer, Paolo Brambilla, Carlo Altamura, Marika Belleri, Francesca Bottinelli, Adele Ferro, Marta Re, Emiliano Monzani, Maurizio Sberna, Giampaolo Perna, Maria Nobile, Alessandra Alciati, Armando D’Agostino, Lorenzo Del Fabro, Matteo Balestrieri, Carolina Bonivento, Giuseppe Cabras, Franco Fabbro, Marco Garzitto, Sara Piccin, Christos Pantelis, Christos Davatzikos

**Affiliations:** 1 Department of Psychiatry and Psychotherapy, University of Lübeck, Lübeck, Germany; 2 Institute for Translational Psychiatry, University of Münster, Münster, Germany; 3 Institute of Psychology, University of Lübeck, Lübeck, Germany; 4 Department of Child Adolescence Psychiatry and Psychotherapy, University of Münster, Münster, Germany; 5 Departmnet of Psychiatry and Psychotherapy, University of Cologne, Faculty of Medicine and University Hospital of Cologne, Cologne, Germany; 6 Department of Psychology and Educational Sciences, Ludwig-Maximilian University, Munich, Germany; 7 Department of Psychiatry, Icahn School of Medicine at Mount Sinai, New York, NY USA; 8 Department of Psychiatry, University of Turku, Turku, Finland; 9 Department of Psychiatry, University of Melbourne & Melbourne Health, Melbourne, Australia; 10 Monash Institute of Pharmaceutical Sciences (MIPS), Monash University, Parkville, VIC Australia; 11 Florey Institute of Neurosciences and Mental Health, Parkville, VIC Australia; 12 Scientific Institute IRCCS “Eugenio Medea”, Pasian di Prato (Ud), Udine, Italy; 13 Department of Psychiatry and Psychotherapy, Medical Faculty, Heinrich-Heine University, Düsseldorf, Germany; 14 Department of Psychology, Faculty of Psychology, Airlangga University, Surabaya, Indonesia; 15 University Hospital of Child and Adolescent Psychiatry and Psychotherapy, University of Bern, Bern, Switzerland; 16 Department of Neurosciences and Mental Health, Fondazione IRCCS Ca’ Granda Ospedale Maggiore Policlinico, Milano, Italy; 17 Department of Pathophysiology and Transplantation, University of Milan, Milan, Italy; 18 Department of Basic Medical Science, Neuroscience and Sense Organs, University of Bari Aldo Moro, Bari, Italy; 19 Department of Psychiatry, University of Oxford, Oxford, UK; 20 Birmingham Early Intervention Service, Birmingham Womens and Childrens NHS Foundation Trust, Birmingham, UK; 21 Institute for Mental Health, University of Birmingham, Birmingham, UK; 22 Department of Radiology, University of Pennsylvania School of Medicine, Philadelphia, PA USA; 23 Institute of Psychiatry, Psychology and Neuroscience, King’s College London, London, UK; 24 Max-Planck-Institute of Psychiatry Munich, Munich, Germany; 25 Department of Psychiatry and Psychotherapy, Ludwig-Maximilian University Munich, Munich, Germany; 26 Department of Psychiatry and Psychotherapy, Ludwig-Maximilian-University, Munich, Germany; 27 Department of Psychiatry and Psychotherapy, University of Cologne, Cologne, Germany; 28 Department of Psychiatry, Psychiatric University Hospital, University of Basel, Basel, Switzerland; 29 Institute for Mental Health and School of Psychology, University of Birmingham, Birmingham, UK; 30 Department of Psychiatry, Psychiatric University Hospital LVR/Heinrich-Heine-University Düsseldorf, University of Düsseldorf, Düsseldorf, Germany; 31 Department of Psychiatry and Psychotherapy, University of Münster, Münster, Germany; 32 GE Global Research, Inc, Munich, Germany; 33 Department of Neuroscience and Mental Health, Fondazione IRCCS Ca’ Granda Ospedale Maggiore Policlinico, Workgroup of Paolo Brambilla, University of Milan, Milan, Italy; 34 Programma 2000, Niguarda Hospital, Workgroup of Paolo Brambilla, University of Milan, Milan, Italy; 35 San Paolo Hospital, Workgroup of Paolo Brambilla, University of Milan, Milan, Italy; 36 Villa San Benedetto Menni, Albese con Cassano, Workgroup of Paolo Brambilla, University of Milan, Milan, Italy; 37 Department of Medical Area, Workgroup of Paolo Brambilla, University of Udine, Udine, Italy; 38 Marco Garzitto and Sara Piccin (IRCCS Scientific Institute E. Medea, Polo FVG, Workgroup of Paolo Brambilla, University of Udine, Udine, Italy; 39 Melbourne Neuropsychiatry Centre, Department of Psychiatry, University of Melbourne & Melbourne Health, Melbourne, Australia; 40 Department of Radiology, University of Pennsylvania School of Medicine, 3700 Hamilton Walk, Philadelphia, PA 19104 USA

**Keywords:** Schizophrenia, Depression

## Abstract

Deeper understanding of Subtle Visual Dysfunctions (VisDys) in the early stage of mental illness and their neurobiological underpinnings, as reflected by microstructural brain texture features, could advance our understanding of the underlying disease perceptual mechanisms that mediate susceptibility to psychosis. In this study, we aim a) to investigate the utility of brain texture features for the prediction of VisDys in recent onset psychosis (ROP) and clinical high-risk syndromes for psychosis (CHR-P), respectively, b) to test prediction models established in ROP and CHR-P in an independent validation sample with recent onset depression (ROD) diagnoses and c) to test for symptom expression related brain features associated with VisDys. sMRI were acquired in a training sample including 128 ROP (67 patients with VisDys), 134 CHR-P (71 patients with VisDys). Independent validation sets included 46 ROP (19 with VisDys), 124 CHR-P (68 patients with VisDys) and a sample of 256 ROD (50 patients with VisDys). Both classification schemas in ROP and CHR-P presented balanced accuracy >77% and >64% in the independent validation samples of ROP, CHR-P, and ROD, respectively. Statistically significant associations were identified with scores from the Positive and Negative Symptom Scale, psychosocial functioning, and the Scale of Negative Symptoms.

## Introduction

Visual dysfunctions (VisDys) are understood as subtle visual perceptual distortions of real features in the environment representing important symptoms of the wide range of perceptual impairments characterizing clinical syndromes of the psychosis spectrum [1–3]. Although such alterations within the visual system are often neglected in clinical practice, they are nonetheless relevant for the understanding of perception-related disease mechanisms in psychosis. The psychosis spectrum includes states of manifest psychotic syndromes such as recent onset psychosis (ROP) but also clinical high risk of psychosis (CHR-P) states, defined by ultra-high risk (UHR) or basic symptom criteria [4] besides more chronic states of psychotic disorders, e.g. recurrent episodes of schizophrenia. Of note, only about 35% of CHR-P with UHR criteria will develop into a diagnosis of manifest schizophrenia within 5 years suggesting that the CHR-P state represents a heterogeneous group in which some subgroups may share neurobiological features with ROP but other subgroups may be characterized by different neurobiological features [5]. Conversion rates for basic symptom criteria, one of which includes VisDys, may be even higher underlining the relevance of VisDys [4]. VisDys, which have first been comprehensively described by Gerd Huber [6], can manifest as perceptual abnormalities [7] regarding perceptual organization [8], contrast sensitivity [9], motion, colour, brightness, and shape including perception of human figures and emotional expressions [10].

Following a recent model, the underlying mechanisms of VisDys are understood as incorrect integration of visual information at early information processing stages within occipital networks [11] resulting in disturbed coding into neuronal signals along different visual streams, e.g. ventral and dorsal visual streams [12], respectively. Interestingly, VisDys at early stages of the disease manifest as rather hypersensitivity to visual stimuli but are suggested to turn into hyposensitivity along illness progression staying nonetheless inconsistent [10]. Previously, VisDys have been related to overt structural lesions in the occipital cortex [13]. Besides this, there is otherwise little known about structural brain tissue alterations, e.g. grey matter loss, related to VisDys. VisDys have also been linked to transdiagnostic vulnerability in mental illness presenting individual differences in aberrant structure [14] and functional connectivity of visual cortical areas of the brain [15]. We recently showed that VisDys [16] were closely related to stronger impairment of functional outcome, increased depression, and poorer quality of life, especially in CHR-P but also ROP.

To expand our previous findings on functional alterations related to VisDys and taking into account the inconsistency of VisDys expression from a long-term perspective, we aim to further explore the neurobiological underpinnings related to VisDys, using structural brain MRI to predict VisDys in ROP and CHR-P from the PRONIA sample. In contrast to volumetric methods, we applied a novel approach that assesses microstructural brain texture characteristics in non-segmented brain sMRI to dive into the molecular level from another perspective which already used in another previous study of our group [17]. More specifically, texture features extracted from non-segmented brain MRI are able to reveal hidden information and explain brain complexity better than existing methods, as these features consider the inter-relations between voxels including those in different modalities (grey/white matter and cerebrospinal fluid). Based on recent own publications on brain texture capability to discriminate mental disorders [17–20], we hypothesized that such radiomic texture feature models capture brain alterations at a microscale level and local spatial organization of grey-level intensities, enabling the prediction of VisDys, compared to macrostructural or network-level differences. Radiomic texture features provide complementary, fine-grained information about local brain tissue integrity, potentially offering higher sensitivity to the transdiagnostic appearance of VisDys. Previous studies describing the VisDys phenomenon using follow-up study designs have pointed out their inconsistency along illness progression [10]. The distinction in the early stages of illness using brain structure data is more challenging, when symptoms and course are more heterogeneous [21]. In the present study, we therefore aimed to identify consistencies in VisDys appearance transdiagnostically across ROP and CHR-P by applying a framework that results on individual level prediction outcomes. We were further interested in the stability of these VisDys associated brain characteristics in relation to symptom load at baseline and after nine months follow-up, validating the predictability of the clinical symptom severity transdiagnostically in patients with and without VisDys, respectively. Expanding the transdiagnostic approach, we were additionally interested in the specificity of VisDys-brain texture associations in the psychosis spectrum compared to recent-onset depression (ROD) patients as an independent validation sample.

## Method

### Study Design

Data were gathered as part of the EU-FP7-funded PRONIA study, a seven-center research project that aimed to optimize the use of potential biomarkers for early diagnosis and prognosis of mental diseases.

### Participants

The general inclusion criteria were as follows: Ages 15 to 40, ability to give informed consent, and language proficiency necessary for participation are the three requirements. General exclusion criteria included any medical indication against MRI, current or past head trauma with loss of consciousness ( > 5 minutes), current or past known neurological or somatic disorders potentially affecting the structure or functioning of the brain, and current or past alcohol dependence and polysubstance dependence within the last 6 months.

The trial was registered with the German Clinical Trials Register (DRKS00005042), and local research ethics boards at each center gave their approval (Ludwig-Maximilian University Munich (ethics ID: 351-13), University of Basel (ethics ID: M12/99), University of Cologne (ethics ID: 13-236), University of Turku (ethics ID: 99/1810/2013), University of Bari (ethics ID: 4754), University of Milan (ethics ID: N.PROT.0001885 | P | GEN/02), University of Udine (ethics ID: 67172), University of Birmingham (ethics ID: 14/WM/0019), University of Münster (ethics ID: 2016-398-b-S) and University of Düsseldorf (ethics ID: 5957 A)), in accordance with the standardized recruitment and assessment protocol from the PRONIA study (**Supplementary S1**). All participants provided written informed consent prior to being included in the study, as did their guardians in the case of underage participants (defined as those under the age of 18 at all locations).

All participants underwent structural MRI (1.5 T and 3 T) [21, 22]. For the current analysis, we used T1-weighted sMRI images of ROP, CHR-P and ROD participants. At every site all images were equally distributed across field strength, visually inspected, automatically defaced and anonymized using an in-house FreeSurfer-based script before the data was centralized. Supplementary Table S1 lists the scanner and parameter details of the structural MR sequences used to examine the PRONIA sample participants. See **Supplementary section 1.1** and previous PRONIA report [21] for full MRI harmonization and data acquisition parameters.

### Training and validation samples

**Fig. 1:**
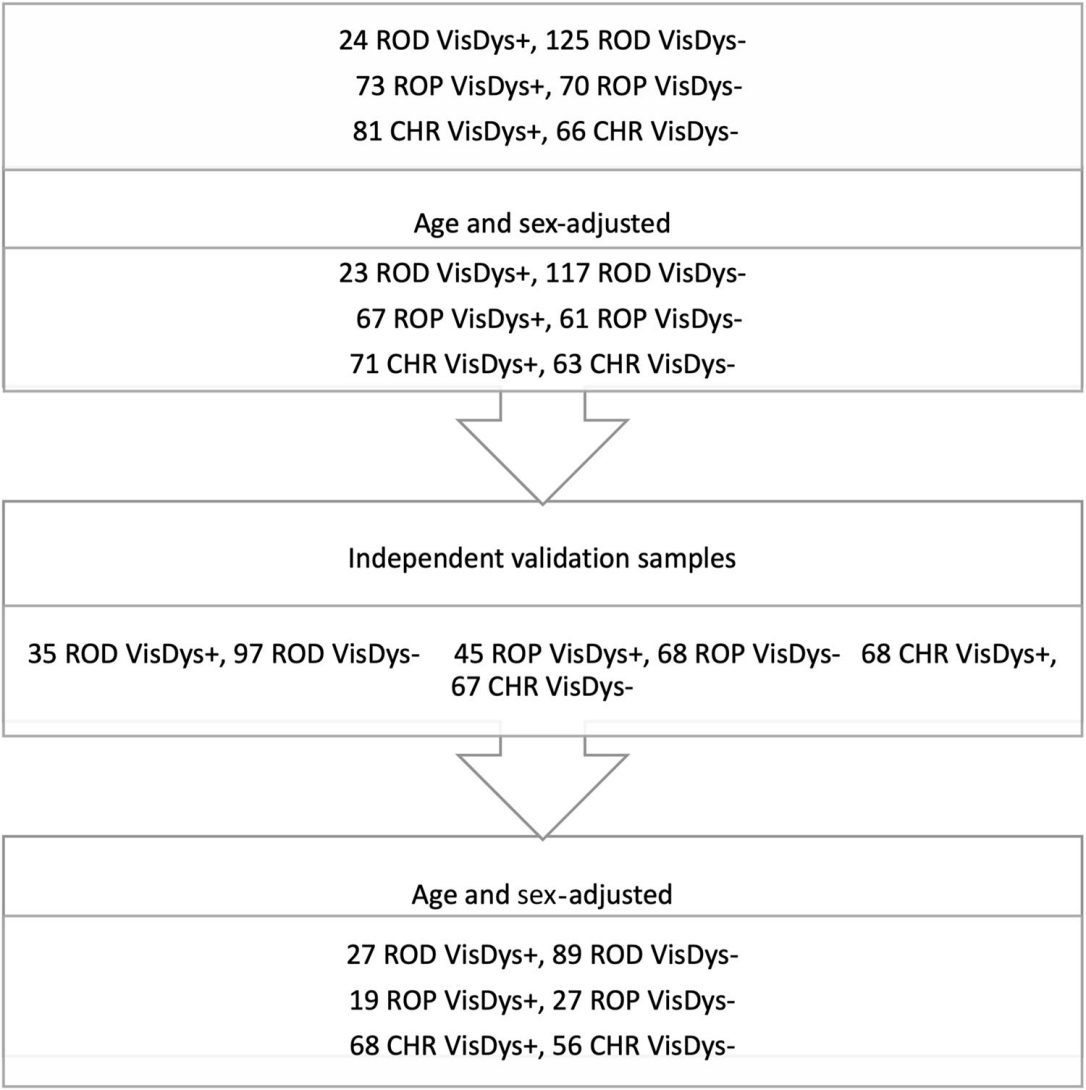
Number of MRIs in the discovery and independent validation samples.

The PRONIA dataset consists of a training sample comprising patients recruited during the first project phase, and the independent validation sample comprising patients recruited during the second project phase. The training sample consisted of brain MRI data from 128 ROP (67 ROP with VisDys (ROP + ), 52.3%, age and sex-adjusted to 61 ROP without VisDys (ROP-)) and 134 CHR-P (71 CHR-P with VisDys (CHR-P + ), 53.0%, age and sex-adjusted to 63 CHR-P without VisDys (CHR-P-)). For independent validation of our models in age and sex-adjusted to samples to the training sample for each group, we used first, 46 ROP (19 ROP + , 41.3%, %, age and sex-adjusted to 27 ROP-) and 124 CHR-P (68 CHR-P + , 54.8%, age and sex-adjusted to 56 CHR-P-) of the PRONIA validation sample, and second, 256 ROD age and sex-adjusted with the training sample (50 ROD with VisDys (ROD + ), 19.5%, age and sex-adjusted to 206 without VisDys (ROD-)) as an independent validation sample (details in Fig. 1).

### Assessments

The following demographic and clinical data—age, sex, and medication exposure—were used in the current analysis: Positive and Negative Symptom Scale (PANSS) [23], the Scale of Negative Symptoms (SANS) [24], the Global Assessment of Functioning (global functioning-role scales) and the Beck Depression Inventory-II (BDI-II) [25]. To capture various facets of VisDys, 14 items were chosen from the Schizophrenia Proneness Instrument, Adult version SPI-A [7, 26]. (see Table S2-S4 in **Supplement file** for more details).

### Analysis

#### Preprocessing

All images were visually inspected, automatically defaced, and anonymized using a Freesurfer-based script prior to data centralization. Subsequently, we used the open-source CAT12 toolbox (version r1155; http://dbm.neuro.uni-jena.de/cat12/), an extension of SPM12 (http://www.fil.ion.ucl.ac.uk/spm), to segment images into grey matter (GM), white matter (WM), and cerebrospinal fluid (CSF) maps and to high-dimensionally register the segmented images to the stereotactic space of the Montreal Neurological Institute coordinates (MNI-152 space) (details in **Supplemental Section 1.1** and Figure S1). We used histogram equalization to adjust the contrast of a greyscale image. The method was applied in a different sample in [18], which concluded in 16 bins. The original image has low contrast, with most pixel values in the middle of the intensity range. The *histeq* function in Matlab produces an output image with pixel values evenly distributed throughout the range and returns a 1-by-256 vector that shows, for each possible input value, the resulting output value (see Figure S1). The number of bins normalizes images and forces the reproducibility of the texture features in new samples [27]. The brain sMRI used have similar resolution and noise levels, a common quantization method, and the same number of grey levels in all quantized images was applied [28, 29]. In this study, we used the *histeq* function with a range of 2 to 256 bins (expressing the number of discrete grey levels), with a step of 2. The optimal number of the bins/bin-width (size) was selected in two stages. First, the images were inspected visually and subsequently selected images were fed into the deep learning pipeline. Very large or small numbers of bins resulted in losing the brain boundaries between GM, WM, and CSF, while extremely noisy images returned. Finally, we exhaustively searched for the optimal number/width of bins by extracting the texture features across all images and feeding them one by one into the deep learning schema. The images with 16 bins returned the highest balanced accuracy. The texture feature maps were extracted from the transformed wp0* image (see Figure S3 for workflow).

#### Feature extraction

Using the 2D grey-level co-occurrence matrix (GLCM) computed in each cube, we were able to extract texture feature maps from non-segmented images. All the feature maps calculated from the 2D GLCM are a function of the probability of each GLCM entry and the difference of the grey levels, *g*_*1*_ and *g*_*2*_ [30]. We calculated texture feature maps only for cubes including non-zero values, as presented in Figure S3 in **the Supplementary file**. We extracted the texture properties of entropy, sum of entropy, difference of entropy, energy, contrast, and homogeneity based on Korda et al. [18]. We focused on the analysis in GLCM-energy and entropy, as these concluded to higher classification accuracies. GLCM energy and entropy capture complementary aspects of texture complexity: energy reflects order and uniformity in spatial grey-level patterns, while entropy quantifies randomness and heterogeneity. Together, they provide a robust measure of local tissue organization and structural integrity. Entropy measures the randomness of the texture distribution, and its inverse measure is the energy, which reflects the regularity and uniformity of the texture distribution, see Figure S2 depicting a representation of energy and entropy feature maps [17].

#### Classification framework

We fed the registered energy and entropy texture feature maps into a 20×20 nested cross-validation deep learning scheme in MATLAB to train and cross-validate models to discriminate VisDys+ and VisDys- in ROP and CHR-P, respectively. See **Supplementary** Figure S4 for further details.

#### Visualization and evaluation of heatmaps

**Fig. 2:**
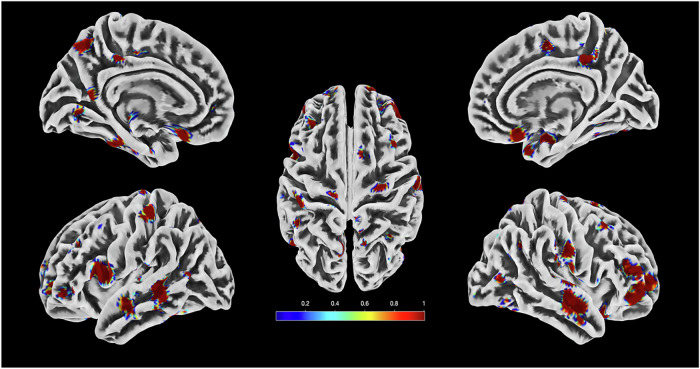
Representation of the voxels that contribute most to the classification decision ROP + /ROD+ based on energy texture features. Different colors represent the fraction of the voxels for each region that are defined as significant.

**Fig. 3:**
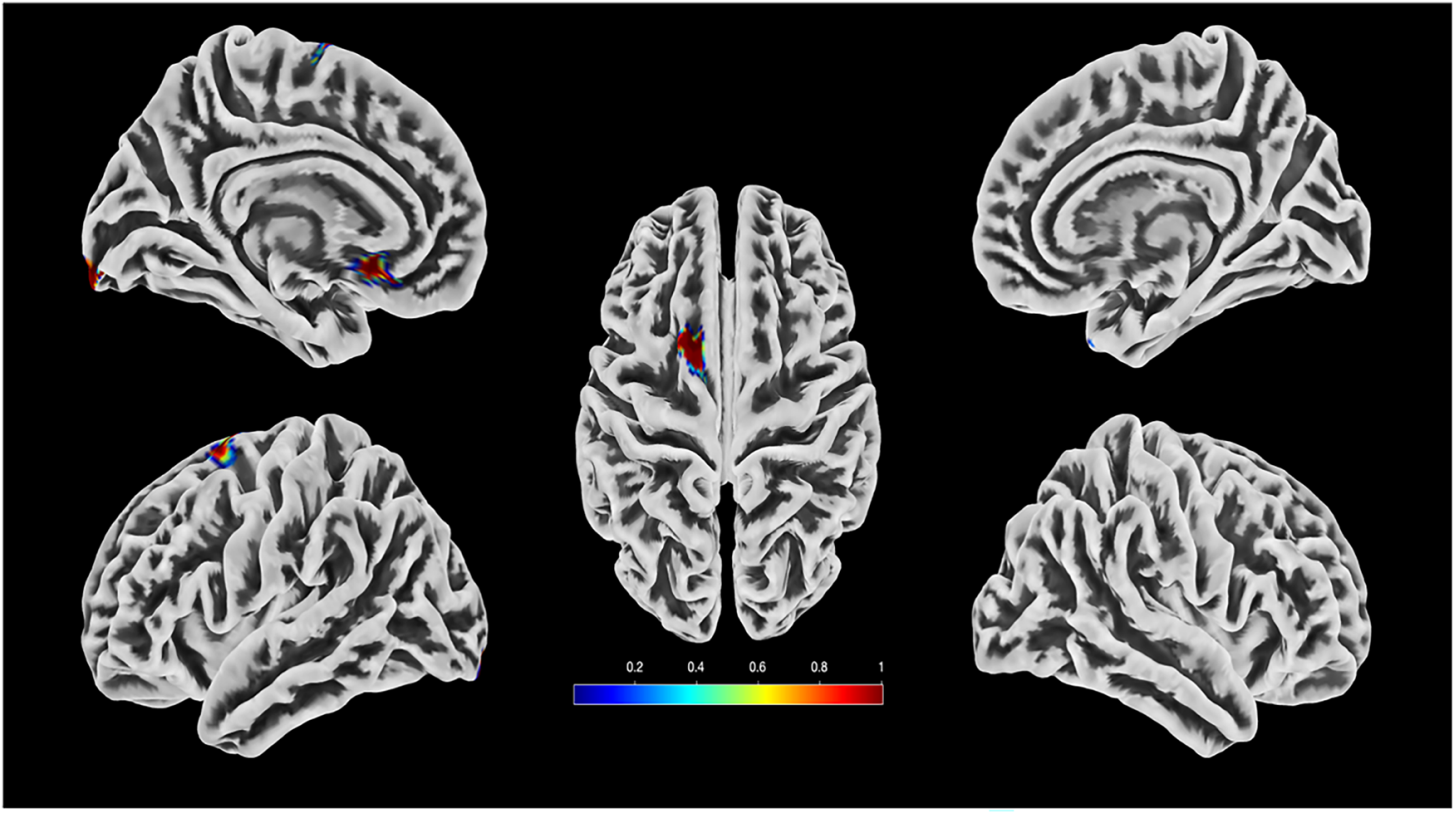
Representation of the voxels that contribute most to the classification decision CHR-P + /ROD+ based on entropy texture features. Different colors represent the fraction of the voxels for each region that are defined as significant.

We used the LRP algorithm for multilayer neural networks, as outlined in Bach et al. [31], to determine the importance of the voxels in each class in order to localize the detected brain alterations in VisDys patients. An individual heatmap that depicts typical alterations in brain structure in general psychopathology is the algorithm’s output (see Appendix A, Supplementary file for further information). Fig. 2 and 3 show visualizations of the classification outcomes for both classification schemas on the holdout dataset.

#### Clustering of subjects and associations with clinical variables

We calculated PANSS factor scores (positive, negative, distress, excitement, and disorganization), the SANS factor scores, psychosocial functioning (GAF), and BDI total scores, as well as VisDys scores at T0. Additionally, we calculated change scores of these variables at T1. We then calculated the Spearman rho coefficients including Bonferroni-Holm correction for multiple comparisons to investigate and model clinical symptom and outcome profiles from the mean brain relevance (heatmap) from whole brain in ROP+ and ROD+ groups in the independent validation at T0 and T1 hold out datasets. Due to heterogeneity of brain texture heatmaps this was not possible in CHR-P+ and ROD+ groups as well as CHR-P- and ROD- groups. We therefore implemented a clustering algorithm to demonstrate shared brain texture patterns across subjects to address group. Our intention was to display the heatmap of each correctly classified subject from the external independent validation sample. The independent validation sample was tested in the winner model of the training sample (see **Supplementary Section 1.4**).

## Results

### Sample characteristics

Sociodemographic and clinical characteristics of the three participant groups with and without VisDys for training (ROP, CHR-P) and validation samples (ROP, CHR-P, ROD) are presented in Table S3. Comparing training and validation samples of ROP there were no statistically significant differences between groups with and without VisDys in clinical characteristics. In CHR-P + , there were statistically significant differences between training and validation samples in GAF, and all PANSS_scores. The CHR- groups from training and validation samples were differentiated by GAF score only. In the training sample, no group differences regarding VisDys-related symptom expression were identified between ROP+ vs. ROP- and CHR-P+ vs. CHR-P-, respectively. Regarding the validation samples, the ROP+ group showed more severe symptom expression in PANSS_negative and PANSS_excitement scores as well as GAF and SANS_anhedonia scores than the ROP- group. Additionally, higher PANSS_positive scores were observed in CHR-P+ compared to CHR-P- from the validation sample. Similarly, higher PANSS_positive scores were observed in the ROD+ compared to the ROD- group.

In Figure S2, it is observed that the energy feature in ROP has higher values in the limit of GM and CSF compared to the entropy in CHR-P that was higher in the outer cortical folding. In ROP, the energy feature was most informative to identify differences between ROP+ and ROP-. Overall, the energy in ROP captures the inner cortex abnormalities and reflects the smoothness of the shape abnormalities, independent of the volume of the brain regions. In CHR-P, the entropy feature was most informative to identify the differences between CHR-P+ and CHR-P-. In CHR-P the entropy reflects the changes of the cortical folding, independent of the volume of the brain regions (see **Supplement** Figure S3 for the whole preprocessing). The high entropy in the outer boundary points reflects a dense microstructural distribution which reflects to changes into cortex as well.

### Classification results and localization

A repeated nested pooled cross-validation classifier of a) **brain energy texture maps** achieved a balanced accuracy (BAC) of 84.85% for discrimination of ROP- against ROP+ in the training sample. Similarly good results were achieved in the independent ROP validation sample with BAC 70.51%, Table 1, and b) **brain entropy texture maps** achieved a BAC of 77.92% for discrimination of CHR-P+ vs. CHR-P- in the training sample. In the independent CHR-P validation sample, this resulted in BAC 64.08%, Table 1. Regarding our third validation sample using the ROD sample, 67.73% of ROD+ were classified correctly by using the energy feature ROP model for the prediction of VisDys. Additionally, 62.32% of ROD+ were also classified correctly using the entropy feature CHR-P model for prediction of VisDys, see Tables 1 and 2. Testing the energy feature model established in ROP by using the energy feature maps established in CHR-P+ vs. CHR-P- resulted in a low BAC of 53.66%, which was even lower compared to ROD energy texture feature maps (BAC 69.36%). This finding suggests common brain texture patterns between ROP and ROD. Similarly, testing the entropy feature maps established in CHR-P using the entropy feature maps of the ROP+ vs. ROP- resulted in lower BACs (BAC 53.45%) compared to ROD entropy texture feature maps (BAC 66.56%).

**Table 1:** Mean classification results for ROP- vs. ROP+ using the energy texture features.

	Balanced Accuracy (%)	Sensitivity (%)	Specificity (%)
ROP-training T0	84.58	85.00	84.17
ROP-external validation T0	70.51	69.69	71.32
Validation with ROD group	69.36	67.73	70.99
Validation with CHR-P group	53.66	43.54	63.82

**Table 2:** Mean classification results for CHR-P- vs. CHR-P+ using the entropy texture features.

	Balanced Accuracy (%)	Sensitivity (%)	Specificity (%)
CHR-P training T0	77.92	75.17	80.67
CHR-P independent validation T0	64.08	53.43	74.72
Validation with ROD group	66.56	62.32	70.86
Validation with ROP group	53.45	32.43	74.46

Implementation of the LRP algorithm showed that voxels with the highest contribution to the prediction of VisDys in ROP and CHR-P (i.e., those with the highest relevance for the classification decision) were located in the frontal and temporal lobes (further details in Figs. 2 and 3 and Supplementary Table S5). The voxels that contributed significantly to the classification decision for the comparison between CHR-P+ vs. CHR-P- were located in frontal and temporal lobes as well as in putamen and caudate.

### Predicting clinical symptom expression at baseline and follow-up by brain texture characteristics

Given the overlap between ROP and ROD regarding energy feature maps related to VisDys on the one hand, and the overlap of entropy feature maps between CHR-P and ROD related to VisDys on the other hand, the associations of the brain relevance, i.e. the voxels that contributed most to the classification decision, with clinical symptom severity at baseline and follow-up were investigated in the combined ROP/ROD validation sample and the combined CHR-P/ROD sample, respectively. We summarize the statistically significant findings here, based on symptom sum scores as described above and outcome profiles.

#### Associations of the brain relevance with symptom expression in patients presenting with VisDys

**Fig. 4:**
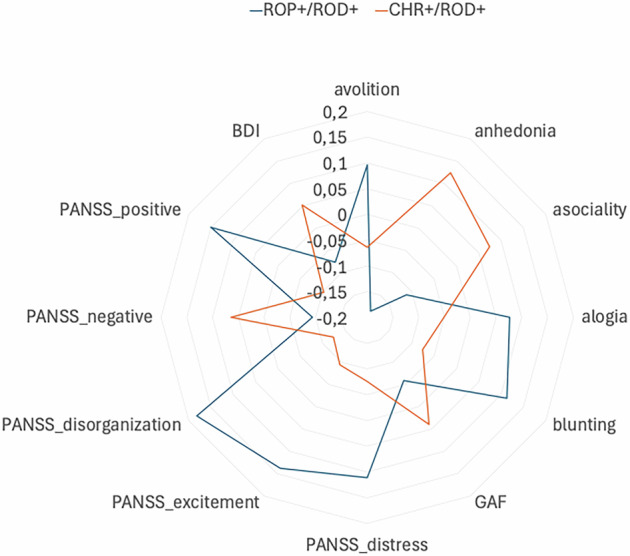
The average values of the clinical variables at T0 for the combined ROP + /ROD+ (blue color) and CHR-P + /ROD+ (orange color) that were used for external validation and further associations.

Using the **energy feature maps**, 44 out of 69 subjects from the combined ROP + /ROD+ groups (from the validation sample) were classified correctly. The average clinical scores are represented in Tables 1B and 1C. Spearman correlation using the mean brain relevance from the **energy feature map** in combined ROP + /ROD+ confirmed the associations with a) PANSS_positive, b) PANSS_disorganization, c) PANSS_excitement scores at baseline (T0), reflected by negative coefficients, i.e. lower brain relevance predicted stronger symptom expression (see Table 3 and Fig. 4). However, GAF score is associated positively with the brain relevance as this is captured from the energy feature. Additionally, change scores after nine-month follow-up (T1) for PANSS_disorganization were predicted with positive coefficients, i.e. higher brain relevance predicted stronger symptom expression change (improvement) over 9 months. (see Table 4). These associations with whole-brain energy features are represented in Figure S5A-E.

**Table 3:** Spearman rho coefficients with p_values corrected by Bonferonni-Holm for the significant associations of whole brain mean relevance heatmaps in ROP + /ROD+ at T0 with PANSS_positive, PANSS_disorganization, PANSS_excitement and GAF (* indicates statistical significant p-values).

Variable	rho	t	P_corrected
PANSS_positive	-0.58	-4.46	0.0010*
PANSS_disorganization	-0.56	-4.29	0.0016*
PANSS_excitement	-0.44	-3.11	0.0412*
GAF	0.47	3.41	0.019*

**Table 4:** Spearman rho coefficients and p_values corrected by Bonferonni-Holm for the significant association of whole brain mean relevance heatmaps in ROP + /ROD+ with the change score from T0 to T1 for PANSS_excitement (* indicates statistical significant p-values).

Variable	rho	t	P_corrected
PANSS_disorganization	0.42	2.39	0.015*

**Fig. 5:**
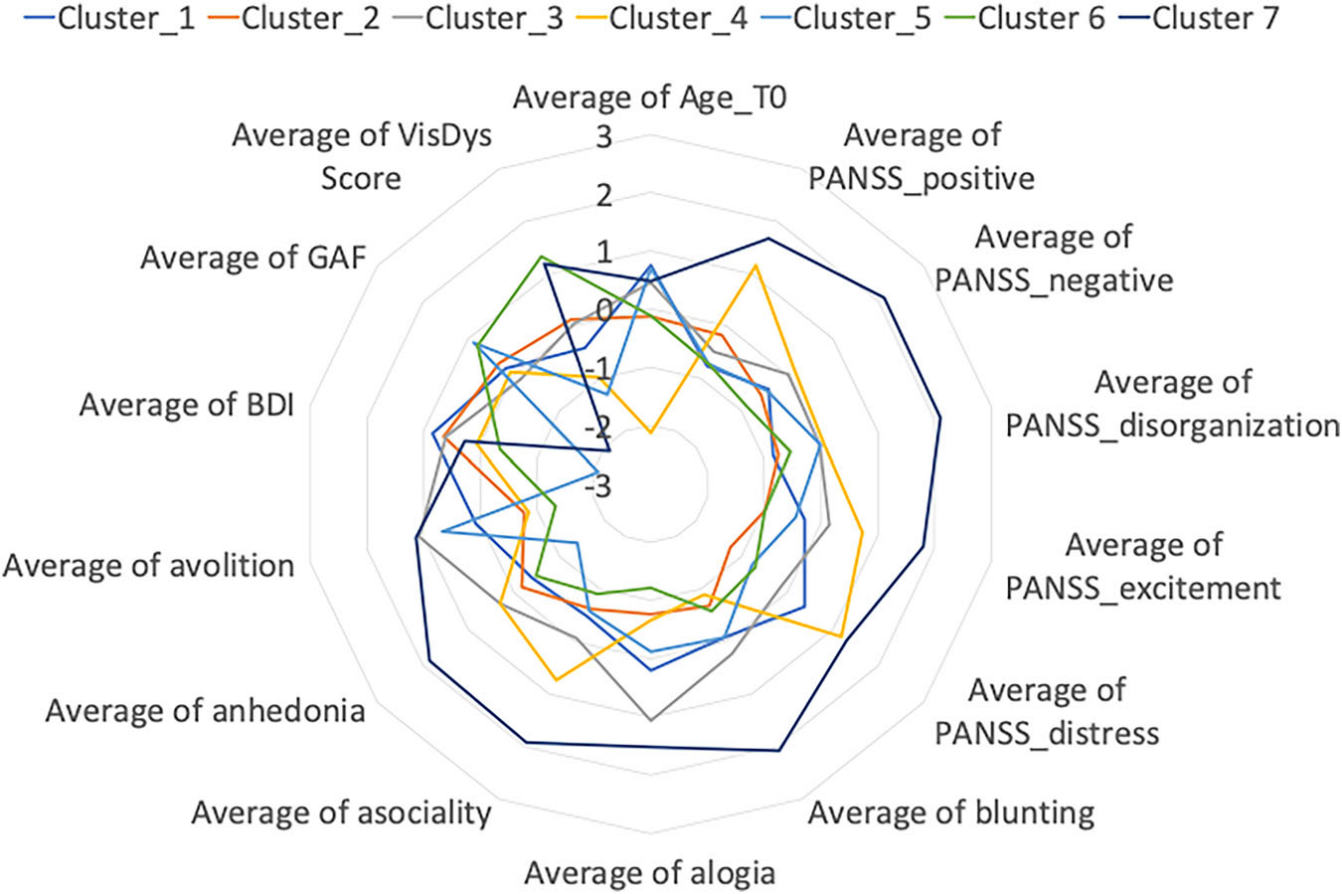
The average values of the clinical variables at T0 calculated in each cluster for the CHR-P+ subjects belong to the independent validation sample and ROD+ subjects. Different colors represent different clusters of brain relevance.

**Fig. 6:**
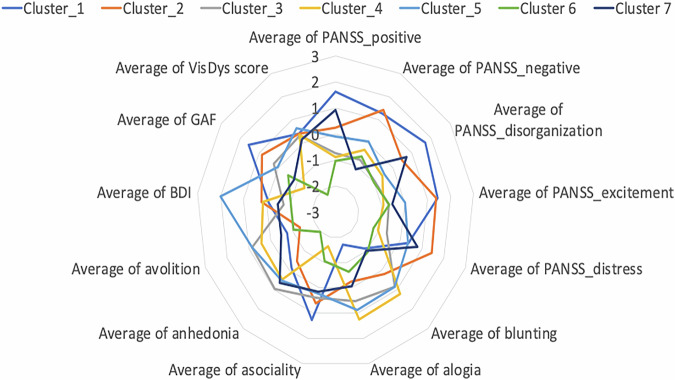
The average values of the difference in symptoms in 9 months after the T0 calculated in each cluster, for the CHR-P+ subjects belong to the independent validation sample and ROD + . Different colors represent different clusters of brain relevance.

Using the **entropy feature map** 62 out of 118 CHR-P + /ROD+ (from the training sample) subjects were classified correctly. Due to the heterogeneity of the CHR-P group, we applied the clustering analysis to the CHR-P/ROD validation samples resulted in 7 clusters (Figs. 5 and 6). On a descriptive level, we found that the highest PANSS and SANS scores were identified for CHR-P+ subjects in cluster 7, with the highest improvement of PANSS_negative after 9 months. The lowest BDI symptoms were grouped in cluster 5, the subjects in this group presented high remission of BDI and VisDys at T1. The lowest GAF was observed in cluster 1 at T0, with high improvement after T1. Cluster 6 presented the highest VisDys score at T0 and the highest improvement of VisDys, PANSS_distress, and anhedonia at T1. Using the mean brain relevance calculated into the clusters from entropy feature maps in combined CHR-P + /ROD+ sample, we can predict the deterioration of PANSS_positive in Cluster 5 (see Table 5, Table S7 and Figure S7F-H). In cluster 5 of the CHR-P + /ROD+ group lower entropy was associated with stronger deterioration of the PANSS_positive score. The subjects in cluster 5 also presented high deterioration of VisDys in the follow-up.

**Table 5:** Spearman rho coefficients and p_values corrected by Bonferonni-Holm for the significant association of whole brain mean relevance heatmaps in CHR-P + /ROD+ with the change score from T0 to T1 for PANSS_positive in cluster 5 (* indicates statistical significant p-values).

Variable	rho	t	P_corrected
PANSS_positive (cluster 5)	-0.93	-5.59	0.033*

#### Association of the brain relevance with symptom expression in patients presenting without VisDys

In ROP-/ROD- there were no associations between whole brain relevance and symptom scores while in CHR-P-/ROD- the AP algorithm resulted in 10 clusters (Figure S6 and S8). The CHR-P-/ROD- group presented higher brain heterogeneity than the CHR-P + /ROD+ group. From the mean brain relevance heatmap in CHR-P-/ROD- we predicted anhedonia and asociality scores (Table S8) in cluster 2 with positive coefficients, i.e. higher brain relevance predicted stronger symptom expression change. Detailed results from groups without VisDys are summarized in Tables in the Supplement.

## Discussion

Our main findings suggest that ROP+ present smoother brain texture shape abnormalities in frontal and temporal lobes than ROP-, as expressed by energy texture feature maps indicating regularity and uniformity of the texture distribution. Energy in ROP reflects inner cortex features which we found to be related to global social functioning in ROP+ indicated by GAF scores. Notably, we found that low energy of the brain in ROP+ predicted low levels of GAF scores. This association gives us a better insight into how energy texture features are related clinically to VisDys, an association which was absent in ROP-. In contrast, CHR-P+ presented sharper grey matter folding of the brain surface in frontal, temporal, and occipital areas as well as higher entropy related to putamen and caudate than CHR-P-, as expressed by entropy texture feature maps indicating the complexity of the texture distribution. Interestingly, we found that in CHR-P + /ROD+ a reduction of PANSS_positive scores from baseline to follow-up was associated with depressive symptom deterioration. Of note, entropy was higher in CHR-P + /ROD+ who present higher BDI score in the follow-up compared to baseline, and this is statistically associated with the improvement of psychotic symptoms. The outer boundary points have been associated with the CHR-P identification compared to ROP in a previous study of Korda et al. [32]. This fact validates the selection of the entropy in this study, as it reflects the boundary folding, which is more dense in CHR than ROP, and especially reflects the presence of VisDys. Due to the heterogeneity of the CHR-P group, we should further investigate the role of the entropy in the outer boundary brain points with the depressive symptom severity. We further show that entropy texture was relevant for the prediction of VisDys in the CHR-P/ROD group underlining the importance of cortical folding changes in the CHR-P+ group compared to ROP+ group. This finding is in line with a previous report from our group in an independent sample showing that cortical boundary complexity is higher in CHR-P compared to ROP and healthy controls [32]. Notably, the resulting significant predictive models from ROP in the present study were not applicable to CHR-P and vice versa, stressing the notion that ROP and CHR-P represent distinguishable mental health conditions related to different neurobiological underpinnings. Besides these specific brain texture features, we also identified common brain patterns across ROP+ and ROD+ groups which were more pronounced than the common brain patterns between CHR-P+ and ROD+ (see sensitivity in Tables 1 and 2). Regarding brain texture features in probands without VisDys, some common brain abnormalities were observed across ROP-, ROD- and CHR-P-, despite a general higher heterogeneity in groups without VisDys than those with VisDys. Occipital and frontoparietal networks in the brain are known as the most relevant to the visual system [10, 33]. From a neurophysiological perspective, VisDys are understood as incorrect integration of visual information at early information processing stages within occipital networks resulting in disturbed coding into neuronal signals along visual streams which are under frontal top-down control. In a previous study on the PRONIA training sample, we focused on these networks to predict VisDys in ROP and CHR-P based on resting state intrinsic brain connectivity, resulting in BACs of 60% in ROP and 67% in CHR-P, respectively [16]. Similarly to the present study, the model derived in the previous study from occipital networks in ROP was not applicable to the occipital network-based CHR-P model, indicating that VisDys in ROP and CHR-P are driven by different alterations in occipital networks. However, a common model across ROP and CHR-P was additionally defined in frontoparietal intrinsic networks, suggesting shared higher-order networks for top-down control of visual information processing across first episode psychosis and clinical high risk state for psychosis [31]. However, follow-up analyses in the previous study identified a subgroup of CHR-P to validate the ROP model supporting the notion that heterogeneity is high in CHR-P regarding susceptibility to psychosis. This is probably also the case in our present results as 53% of CHR-P+ were classified correctly using the ROP model.

Our present findings using texture features from structural brain images, in contrast to intrinsic brain connectivity, go beyond these previous findings [16], first, by revealing more robust and higher accuracies with equally high specificity and sensitivity for the prediction of VisDys in both ROP and CHR-P to a small-scale, almost to the molecular level. These models were validated in independent samples, including also a group of ROD. Note, although VisDys occur much less frequently in ROD than ROP and CHR-P, they may nonetheless indicate a certain neurobiologically based susceptibility to psychosis in individuals presenting with a depressive syndrome [17]. However, in this study, the main goal was to investigate the association of the VisDys transdiagnostically with clinical symptom severity and outcome profile. Second, in line with our previous study, we found frontoparietal areas to be involved in the prediction of VisDys in all groups, while occipital areas were mainly identified in CHR-P and ROD. This finding is in line with other findings from our group using radiomics texture features unrelated to VisDys, in which CHR-P presented statistically significant differences from ROP in the occipital lobe [11, 18]. Furthermore, in the present study, we found parts of the default mode and dorsal network to contribute to the prediction of VisDys in ROP. In ROP the superior longitudinal fasciculus was involved, which is critically involved in visual and spatial cognition [34]. In CHR-P, the middle cerebellar peduncle and other parts of it, which convey information related to eye movement [35], corpus callosum related to unconscious vision [36] and other regions related to involuntary movements were involved. We here show that ROP presenting with VisDys were specifically impaired regarding their remission by identifying homogeneous brain texture changes, which were highly associated with PANSS_positive, disorganization, and excitement scores at T0 and the difference between T1 and T0, interestingly. Our present findings further support the observations from another previous study in which texture features analyses were derived from contrast images but the aspect of VisDys was neglected [17]. Similarly, in the present study we identified contrast feature clusters which contributed significantly to the classification decision, and which predicted accurately the PANSS and GAF scores in a combined ROP + /ROD+ group.

More specifically, in our previous paper [17], brain relevance was associated with PANSS_positive score in ROP and ROD, which is also in line with our present findings in ROP + /ROD + . These facts confirm that different texture features relate to different diagnoses, i.e. the contrast feature can predict outcomes in ROP/ROD [17], but the VisDys in ROP/ROD are identified using the energy feature. However, only ROP + /ROD+ were negatively associated with PANSS scores at T0 (positive, disorganization and excitement) and positively with GAF score, which means that the lower the energy, i.e. the smoother the brain shape abnormalities at baseline, the stronger is symptom expression in patients with VisDys and disorganization scores at nine-month follow-up at T1, but lower the functionality at T0. Thus, low brain texture energy can predict worse outcome, especially related to the aspect of disorganization. We observed a similar constellation in cluster 5 of the CHR-P + /ROD+ group in which subjects with low BDI score and deterioration of the BDI and VisDys score in follow-up, were associated negatively with the PANSS_positive score. This means that the sharpness of the cortical folding was associated negatively with the PANSS_score in patients with low BDI and high VisDys score. In CHR-P and ROD group the oldest subjects were grouped together and presented a deterioration of PANSS scores at follow-up in both groups with or without VisDys. Also, independent of the existence of VisDys in CHR-P and ROD group the subjects with low BDI presented a deterioration of the BDI at follow-up. This is a confound of the early stage of the illness in older people and it is not related to the VisDys. In CHR-P + /ROD + , cluster 5, the highest the sharpness of the brain surface folding (entropy) the lower the PANSS_positive in patients with very low BDI which lead to worsening of VisDys in the follow-up. In addition, the change in anhedonia and asociality scores were all positively associated with brain entropy (energy) in young depressive CHR-P-/ROD-.

Our present findings with respect to CHR-P are in line with our previous report from VisDys releated functional connectivity, where we found that VisDys were associated with a broader range of symptoms including impairments of quality of life, depressive symptom expression and neurocognitive impairments, i.e. visuospatial constructability, in addition to functional impairments [16]. Similarly, our present findings reflect a higher clinical heterogeneity in this group than in the ROP + /ROD+ group. This constellation of findings with less clearly described symptom expression in CHR-P + /ROD+ compared to ROP + /ROD+ may parallel the observation that VisDys at early illness stages manifests as rather hypersensitivity reflecting a still unstable state but turning into hyposensitivity along illness progression, staying nonetheless inconsistent [10].

To summarize, the present study also suggests that VisDys in ROP are driven by more sustained structural alterations in occipito-fronto-parietal networks while VisDys in CHR-P form a more heterogeneous group related to more heterogeneous, inconsistent neurobiological underpinnings. These findings are depicted to the clinical symptom profiles as well; different brain regions across diagnoses for patients with or without VisDys were associated differently with the clinical symptom severity and outcome profile, generating proof-of-concept homogeneous clusters of symptoms. Our findings further suggest that within CHR-P specific VisDys-related subgroups can be identified that are closer to ROP than other CHR subgroups.

### Limitations

At the moment, neither a distinct biological etiology nor underlying brain structures serve as the basis for VisDys underpinning in early psychotic states. The cross-sectional and longitudinal character of our dataset must be well understood when interpreting the results, since this is crucial because there may be dynamic and changing symptom cluster profiles that are missed. The variation in MRI intensity standardization impacts the texture feature extraction. Although the validation sample and the training sample were both drawn from the same research, it is crucial to remember that not all of the validation sample’s patients were drawn from the same locations as the discovery patients. Environmental and ethnicity factors may affect the prediction accuracies; however, the main goal of the study is to investigate the VisDys manifestation transdiagnostically using brain sMRI. Due to this constraint, we stress that more validation in bigger and geographically diverse cohorts is required, and that the current results are a proof-of-concept. The model’s generalizability and stability as well as its use in clinical practice should be carefully examined in future research and tested in larger samples which include information on brain texture and function together with VisDys assessments.

## Conclusions

Together, our findings are in line with previous reports of grey and white matter abnormalities in patients with severe mental syndromes, forming specific VisDys-related brain texture signatures. The energy and entropy are state-specific, changing with the severity of symptoms, e.g., psychotic symptoms and functional outcome. This observation, along with the study’s proof-of-concept symptom findings, bolsters our hypothesis that neuroimaging biomarkers might be helpful in predicting outcome profiles related to VisDys transdiagnostically. The establishment of different clusters that are connected to VisDys and outcome profiles represents this study’s novel discovery.

## Supplementary information

Supplementary File

## Data Availability

The code has been implemented in the PRONIA serverand extracted features are available upon request.

## Data availability

The datasets used and/or analysed during the current study will be available from the authors upon reasonable request submitted to the PRONIA Consortium.
