# Supplementary material for: Brain texture alterations predict subtle visual perceptual dysfunctions in recent onset psychosis and clinical high-risk state
Source: Transl Psychiatry. 2026 Feb 12;16:113. doi: 10.1038/s41398-026-03840-x (PMC12923831; doi:10.1038/s41398-026-03840-x)
Supplement: Supplementary file 1 — Supplementary File [file 41398_2026_3840_MOESM1_ESM.docx]

**Supplementary file**

**Table S1:** MR scanner systems and structural MRI sequence parameters used at the respective PRONIA sites.

| **PRONIA Site** | **Model** | **Field Strength** | **Coil Channels** | **Flip Angle** | **TR [ms]** | **TE [ms]** | **Voxel Size [mm]** | **FOV** | **Slice Number** |
| --- | --- | --- | --- | --- | --- | --- | --- | --- | --- |
| Munich | Philips Ingenia | 3T | 32 | 8 | 9.5 | 5.5 | 0.97 x 0.97 x 1.0 | 250 x 250 | 190 |
| Milan Niguarda | Philips Achieva Intera | 1.5T | 8 | 12 | Shortest (8.1) | Shortest (3.7) | 0.93 x 0.93 x 1.0 | 240 x 240 | 170 |
| Basel | SIEMENS Verio | 3T | 12 | 8 | 2000 | 3.4 | 1.0 x 1.0 x 1.0 | 256 x 256 | 176 |
| Cologne | Philips Achieva | 3T | 8 | 8 | 9.5 | 5.5 | 0.97 x 0.97 x 1.0 | 250 x 250 | 190 |
| Birmingham | Philips Achieva | 3T | 32 | 8 | 8.4 | 3.8 | 1.0 x 1.0 x 1.0 | 288 x 288 | 175 |
| Turku | Philips Ingenuity | 3T | 32 | 7 | 8.1 | 3.7 | 1.0 x 1.0 x 1.0 | 256 x 256 | 176 |
| Udine | Philips Achieva | 3T | 8 | 12 | Shortest (8.1) | Shortest (3.7) | 0.93 x 0.93 x 1.0 | 240 x 240 | 170 |

In keeping with real-world scanner heterogeneity and as part of the larger PRONIA goals, the PRONIA sites were required to (1) acquire isotropic or nearly isotropic voxel sizes of at least 1 mm resolution, (2) set the Field of View (FOV) parameters accordingly to ensure full 3D coverage of the brain, including all parts of the cerebellum, and (3) define the relaxation time (TR) and echo time (TE), as well as other ionizing radiation parameters.

In brief, both inpatient and outpatient services were used to recruit individuals. A uniform protocol for recruitment and assessment was followed. Following the index determination, the observational research protocol called for re-exams every three months over an overall follow-up of 9 months. To guarantee that the Positive and Negative scale for Schizophrenia (PANSS scores) and Global Functioning (GAF) measures were reliable across research sites, regular interrater reliability tests were conducted.

The prerequisites for ROP participants were a first episode of affective or nonaffective psychosis according to the Structured Clinical Interview for DSM-IV-TR (SCID)^1^ fulfilled within past 3 months and an onset not more than 24 months ago. The subsequent specific ROP exclusion criteria included the onset of psychosis more than 24 months prior and the use of antipsychotics for more than 90 days (cumulatively in the previous 24 months) with a daily dose rate at or above the German Society for Psychiatry, Psychotherapy, and Nervous Diseases (DGPPN) S3 guidelines' "First-Episode Psychosis" range minimum dosage, which is equivalent to 5 mg of olanzapine.

It was necessary for patients with ROD to satisfy a major depression criteria set out by SCID, which they had to have done within the preceding three months. Specific ROD exclusion criteria included the following: the current episode's length of more than 24 months; the cumulative use of antipsychotic medication for more than 30 days; any intake of antipsychotic drugs within the past 3 months before psychopathological baseline assessments at or above the minimum dosage threshold; and any DSM-IV-TR major depressive episode that preceded the current or recent episode. Patients meeting CHR-P criteria according to the SIPS and/or the basic symptom criterion Cognitive Disturbances were also excluded from the ROD group. Clinical raters received training in the assessment of clinical high-risk criteria for psychosis (CHR-P), including both Ultra High Risk and COGDIS symptom assessment instruments^2^ through workshops provided by author FSL, the developer of the Schizophrenia Proneness Instrument (SPI). CHR-P criteria of each study candidate were assessed before study inclusion in regular case conferences led by FSL and re-evaluated during the follow-up period if a transition to psychosis was suspected. Individuals meet CHR criteria if they exhibit one or more of the following symptoms: (1) A schizotypal personality disorder or a first degree relative with psychosis plus a recent decline in function, (2) ‘Attenuated’ positive psychotic symptoms, like ideas of reference, odd beliefs, magical thinking, or unusual perceptual experiences, and/or (3) A brief psychotic episode of less than one week duration that resolves without antipsychotic medication.

SPI-A variables were coded in the following way: [0 (absent)=0), 1(rare)=1, 2 (mild)=2, 3 (moderate)=3, 4 (moderately severe)=4, 5 (severe)=5, 6 (extreme)=6, 7(trait)=0, 8 (present but severity unknown)=1, 9(questionable)=0], to determine the severity of each VisDys based on participants’ reports. As previously reported ^3^ a sum score was calculated for each participant individually over the 14 SPI-A elements. For additional analyses, participants were then divided into two groups, VisDys+ (sum score>0) and VisDys–(sum score=0), respectively, based on this sum score Raters had been trained in the SPI-A by co-author FSL.

We calculated five subscores of PANSS (positive, negative, disorganized, excitement, distress) based on the factor analysis by Wallwork et al.^4^. Several factor-analytic studies have suggested that a five-factor model better captures PANSS structure in schizophrenia samples^4^. For the Global Assessment of Functioning (GAF), we used ratings based exclusively on level of functioning GAF-d; symptoms were not considered. SANS factor scores provide a more detailed and differentiated picture of negative symptoms, some of which are not included in the negative factor PANSS, while others may have varying courses. In **Table S3**, the mean and standard deviation of scores of ROP, CHR-P and ROD from the training sample (A) and the independent validation sample (B) are presented. We compared between the same patient groups with and without VisDys using independent Mann-Whitney U-tests.

**Table S2:** Intra-class correlation analysis of the GAF:S / GAF:R scores generated by the PRONIA raters on the test cases.

|  | **Intra-class correlation analysis** of the GAF:S / GAF:R scores generated by the PRONIA raters on the test cases. **PRE-TRAINING ICCs**  For all Sites  (95% CI) | **POST-TRAINING ICCs**  For all sites  (95% CI) |
| --- | --- | --- |
| Social & Role | **.836**  (.751 - .910) | **.871**  (.801 - .931) |

Andrea Auther (AA), one of the authors of the GAF and PANSS scales, tested the PRONIA consortium independently using four written transcripts of interviews conducted at Zucker Hillside Hospital. She provided repeated training to PRONIA investigators. To assess the between-rater agreement on the target measures, thirty-six PRONIA raters conducted an Intra Class Correlation (ICC) analysis. Raters were required to produce six functional scores for each reliability test, including the PANSS scores and the social and role functioning domain (GAF:S/GAF:R) scores—the current, lowest, and highest in the previous year. **Supplemental Table S2** displays the GAF scores' ICC analysis results. The following criteria for interpreting kappa or ICC inter-rater agreement measures are provided by Cicchetti 3: Less than 0.40 denotes poor quality, between 0.40 and 0.59 fair, between 0.60 and 0.74 good, and between 0.75 and 1.00 excellent. Each participant's psychopathology was evaluated by qualified clinicians, and interrater reliability tests were conducted on a regular basis to calibrate PANSS [(ICC)=0.79] across research sites. There is no available interrater reliability test for SANS.

- 1. **MRI processing**

The PRONIA consortium aimed to generate an MRI database that would represent the MR scanner sequence heterogeneity encountered in clinical real-world. Therefore, the project included a calibration study using the sMRI images of six healthy travelling volunteers who were scanned at all sites with the same parameters^5^. Processing steps applied to the structural images, consisting of (1) the 1^st^ denoising step based on Spatially Adaptive Non-Local Means (SANLM) filtering ^6^;(2) an Adaptive Maximum A Posteriori (AMAP) segmentation technique, which models local variations of intensity distributions as slowly varying spatial functions and thus achieves a homogeneous segmentation across cortical and subcortical structures^7^; (3) the 2^nd^ denoising step using Markov Random Field approach which incorporates spatial prior information of adjacent voxels into the segmentation estimation generated by AMAP^7^; (4) a Local Adaptive Segmentation (LAS) step, which adjusts the images for white matter (WM) inhomogeneities and varying gray matter (GM) intensities caused by the differing iron content in e.g. cortical and subcortical structures. The LAS step is carried out before the final AMAP segmentation; (5) a partial volume segmentation algorithm that is capable of modeling tissues with intensities between GM and WM, as well as GM and cerebrospinal fluid (CSF) and is applied to the AMAP-generated tissue segments; (6) a high-dimensional DARTEL registration of the image to a MNI-template generated from the MRI data of 555 healthy controls in the IXI database (http://www.braindevelopment.org). The registered GM images were multiplied with the Jacobian determinants obtained during registration to produce GM volume (GMV) maps. Images were smoothed with 10 mm before entering the subsequent analysis steps. (8)The Quality Assurance framework of CAT12 was used to check the quality of the GMV maps. The pre-processing steps were the same for all the PRONIA datasets and were executed on the Ludwig-Maximilian University Munich (LMU) server by Prof. Nikolaos Koutsouleris. Authors had access to the processed data, i.e., the preprocessed non-segmented brain MRI.

**Table S3:** Descriptives of the SPI-A 14 items for the Validation sample

|  |  | | | **Validation Sample** | | | | |  |
| --- | --- | --- | --- | --- | --- | --- | --- | --- | --- |
| **SPI-A item** | **Group** | | | **Mean** | | **SD** | | |  |
| Oversensitivity to light/visual  objects |  | ROP | 0.8328 | | 1.846 | |  |  | |
|  |  | CHR-P | 1.0109 | | 1.917 | |  |  | |
|  |  | ROD | 0.2862 | | 1.062 | |  |  | |
| Photopsia |  | ROP | 0.4332 | | 1.283 | |  |  | |
|  |  | CHR-P | 0.3201 | | 1.038 | |  |  | |
|  |  | ROD | 0.0986 | | 0.576 | |  |  | |
| Micropsia/macropsia |  | ROP | 0.1238 | | 0.670 | |  |  | |
|  |  | CHR-P | 0.1403 | | 0.754 | |  |  | |
|  |  | ROD | 0.0179 | | 0.198 | |  |  | |
| Near and tele-vision |  | ROP | 0.2361 | | 1.005 | |  |  | |
|  |  | CHR-P | 0.2446 | | 0.960 | |  |  | |
|  |  | ROD | 0.0215 | | 0.223 | |  |  | |
| Metamorphopsia |  | ROP | 0.1902 | | 0.927 | |  |  | |
|  |  | CHR-P | 0.0935 | | 0.581 | |  |  | |
|  |  | ROD | 0 | | 0 | |  |  | |
| Changes colour vision |  | ROP | 0.2778 | | 1.058 | |  |  | |
|  |  | CHR-P | 0.295 | | 1.104 | |  |  | |
|  |  | ROD | 0.0394 | | 0.435 | |  |  | |
| Changed perception of own face |  | ROP | 0.2745 | | 1.019 | |  |  | |
|  |  | CHR-P | 0.3129 | | 1.064 | |  |  | |
|  |  | ROD | 0.0179 | | 0.158 | |  |  | |
| Pseudomovements optic stimuli |  | ROP | 0.3039 | | 1.117 | |  |  | |
|  |  | CHR-P | 0.3610 | | 1.103 | |  |  | |
|  |  | ROD | 0.0323 | | 0.230 | |  |  | |
| Diplopia |  | ROP | 0.2222 | | 0.966 | |  |  | |
|  |  | CHR-P | 0.1187 | | 0.677 | |  |  | |
|  |  | ROD | 0.0322 | | 0.383 | |  |  | |
| Estimation of distances/sizes |  | ROP | 0.2680 | | 1.053 | |  |  | |
|  |  | CHR-P | 0.1877 | | 0.817 | |  |  | |
|  |  | ROD | 0.0394 | | 0.418 | |  |  | |
| Perception of straight lines/contours |  | ROP | 0.2059 | | 1.011 | |  |  | |
|  |  | CHR-P | 0.1511 | | 0.735 | |  |  |  |
|  |  | ROD | 0.0179 | | 0.299 | |  |  |  |
| Maintenance of optic stimuli |  | ROP | 0.2059 | | 0.913 | |  |  |  |
|  |  | CHR-P | 0.2122 | | 0.912 | |  |  |  |
|  |  | ROD | 0.0753 | | 0.528 | |  |  |  |
| Tubular vision |  | ROP | 0.2549 | | 1.108 | |  |  |  |
|  |  | CHR-P | 0.1367 | | 0.708 | |  |  |  |
|  |  | ROD | 0.0251 | | 0.274 | |  |  |  |
| Captivation by visual details |  | ROP | 0.448 | | 1.348 | |  |  |  |
|  |  | CHR-P | 0.2986 | | 1.051 | |  |  |  |
|  |  | ROD | 0.0681 | | 0.456 | |  |  |  |
|  |  |  |  |  |  |  |  |  |  |

**Table S4:** Clinical and demographic characteristics (mean and standard deviation) for the training samples ROP and CHR-P (A), and external validation samples for ROP and ROD (B) and ROD (C) at T0.

1. **ROP and CHR-P training samples**

| **Group** | **ROP-** | **ROP+** | | **CHR-P-** | | **CHR-P+** | |  |
| --- | --- | --- | --- | --- | --- | --- | --- | --- |
| Number | 61 | 67 | | 63 | | 71 | |  |
| Age mean | 25.6(5.72) | 25.5(5.41) | | 23.9(4.94) | | 23.3(4.82) | |  |
| Sex *n* (%) male | 38 (56.7%) | 37(43.5%) | 27 (45.7%) | | 41(57.7%) | |  | |
| VisDys Score | - | 8.43(10.6) | - | | 7.29(6.58) | |  | |
| GAF | 45.8 (13.7) | 43.8(11.2) | 58.22 (14.71) | | 53.87(12.47) | |  | |
| PANSS_positive | 21.3(7.25) | 21.8(6.55) | 12.53 (5.10) | | 13.35(3.94) | |  | |
| PANSS_negative | 16.8(8.40) | 18.3(9.64) | 13.93 (7.42) | | 14.33(7.22) | |  | |
| PANSS_excitement | 15.1(5.73) | 15.4(5.39) | 11.73 (4.16) | | 11.75(3.71) | |  | |
| PANSS_distress | 19.9(7.61) | 21.6(7.17) | 17.08 (5.48) | | 17.14(5.02) | |  | |
| PANSS_disorganization | 21.06(9.17) | 21.07(8.29) | 14.47 (5.04) | | 14.72(4.19) | |  | |
| SANS_Blunted Affect | 0.357 (0.83) | 0.727(1.39) | 0.59(0.91) | | 0.63(0.89) | |  | |
| SANS_Alogia | 0.780 (1.17) | 0.750(1.16) | 0.63(0.91) | | 0.75(1.02) | |  | |
| SANS_Avolition | 0.395 (1.10) | 0.688(1.31) | 0.44(0.73) | | 0.71(0.96) | |  | |
| SANS_Anhedonia | 2.52 (1.96) | 2.90(1.86) | 1.69 (1.85) | | 2.25(1.75) | |  | |
| SANS_Asociality | 0.750 (1.17) | 1.18(1.26) | 0.78 (0.90) | | 0.98(1.03) | |  | |
| BDI-II | 18.7 (13.3) | 19.3(12.3) | 21.88 (12.77) | | 25.72(14.08) | |  | |

Note: ROD, recent onset depression; ROP, recent onset psychosis; CHR-P, clinical high risk; PANSS, Positive and Negative Symptom Scale; BDI-II, Beck Depression Inventory-II, SANS, GAF

*^a^: Mann-Whitney U-tests showed statistically significant differences (p<0.05) between patients with and without VisDys*

1. **ROP and CHR-P independent validation samples**

| **Group** | **ROP-** | **ROP+** | **CHR-P-** | **CHR-P+** | |  |
| --- | --- | --- | --- | --- | --- | --- |
| Number | 27 | 19 | 56 | 68 | |  |
| Age | 25.3(5.47) | 28.2(4.24) | 25.85(5.85) | 22.41(5.03) | |  |
| Sex *n* (%) male | 27 (100%) | 19 (100%) | 24 (42.9%) |  | 29(42.6%) |  |
| VisDys Score | - | 8.53(10.11) |  | - | 7.54 (7.86) |  |
| GAF | 45.36 (14.99) | 41.05 (15.02) | 49.01 (10.63) |  | 46.82 (11.22) |  |
| PANSS_positive | 21.05 (7.03) | 22.32 (7.44) | 13.06^a^ (4.62) |  | 15.94^a^ (5.18) |  |
| PANSS_negative | 17.00 (9.90) | 15.26 (5.89) | 16.42 (7.51) |  | 18.86 (9.00) |  |
| PANSS_excitement | 15.14 (6.09) | 15.47 (4.49) | 12.74 (4.09) |  | 14.28 ^a^ (4.42) |  |
| PANSS_distress | 20.0 (6.68) | 19.63 (6.15) | 18.69 (6.86) |  | 20.59 (6.02) |  |
| PANSS_disorganization | 22.22 (9.21) | 19.53 (6.36) | 15.47 (5.55) |  | 17.54 ^a^ (6.37) |  |
| SANS_Blunted Affect | 0.56 (1.33) | 0.20 (0.45) | 0.85 (1.08) |  | 1.24 (1.30) |  |
| SANS_Alogia | 0.64 (1.01) | 0.78 (1.09) | 0.99 (1.23) |  | 1.08 (1.24) |  |
| SANS_Avolition | 0.38 (1.09) | 0.30 (0.67) | 0.67 (0.96) |  | 0.73 (1.13) |  |
| SANS_Anhedonia | 2.09 (1.82) | 2.42 (1.54) | 1.69 (1.85) |  | 2.75 ^a^ (1.72) |  |
| SANS_Asociality | 0.64 (0.92) | 1.00 (1.00) | 1.15 (1.24) |  | 1.62 (1.23) |  |
| BDI-II | 11.74 (12.99) | 20.58 (14.93) | 21.21 (13.54) |  | 25.72 (14.27) |  |

*^a^: Mann-Whitney U-tests showed statistically significant differences (p<0.05) between patients with and without VisDys*

**C. ROD validation sample**

| **Group** | **ROD-** | | | **ROD+** | | | | | | |  |  |  |  |
| --- | --- | --- | --- | --- | --- | --- | --- | --- | --- | --- | --- | --- | --- | --- |
| Number | 206 | | | 50 | | | | | |  |  |  |  |  |
| Age mean | 25.9 (6.18) | | | 25.41 (6.18) | | | | | |  |  |  |  |  |
| Sex *n* (%) male | | 88 (42.7%) | | | 31 (62%) | | | |  | | | |  |  |
| VisDys Score | - | | | 4.02 (3.42) | | | |  | |  | |  |  |  |
| GAF | 54.66 (14.62) | | | 55.06 (14.56) | | | | | |  | |  |  |  |
| PANSS_positive | | | 9.53 ^a^ (2.42) | | | | 11.31 (3.31) | | | | | | |  |
| PANSS_negative | | | 14.81 ^a^ (6.42) | | | | 16.71 (6.48) | | | | | | |  |
| PANSS_excitement | | | 11.55 (3.43) | | | | 12.47 (4.31) | | | | | | |  |
| PANSS_distress | | | 17.58 (5.58) | | | | 18.25 (7.09) | | | | | | |  |
| PANSS_disorganization | | | 12.93 (3.01) | | | | 13.76 (4.24) | | | | | | |  |
| SANS_Blunted Affect | | | 0.52 ^a^ (0.79) | | | | 0.79 (0.87) | | | | | | |  |
| SANS_Alogia | | | 0.52 ^a^ (0.79) | | | 0.86 (0.99) | | | | | | | |  |
| SANS_Avolition | | | 0.25 ^a^ (0.55) | | | 0.54 (0.75) | | | | | | | |  |
| SANS_Anhedonia | | | 2.26 (1.85) | | | 2.16 (1.91) | | | | | | | |  |
| SANS_Asociality | | | 1.27 (1.19) | | | 1.31 (1.25) | | | | | | | |  |
| BDI-II (SD) | | | 24.73 (14.85) | | | 27.42 (12.91) | | | | | | | |  |

*^a^: Mann-Whitney U-tests showed statistically significant differences (p<0.05) between patients with and without VisDys*

In **Figure S1**, we have plotted the transformation curve for histogram equalization, the initial intensities and the adjusted intensities and the difference in the intensity between the initial and the transformed image is presented.

**Figure S1:** Representation of a) the transformation curve for histogram equalization and b) the adjusted intensities using histogram equalization, c) the brain MRI in SPM12 for the initial MR image and b) transformed MR image using the histogram equalization.


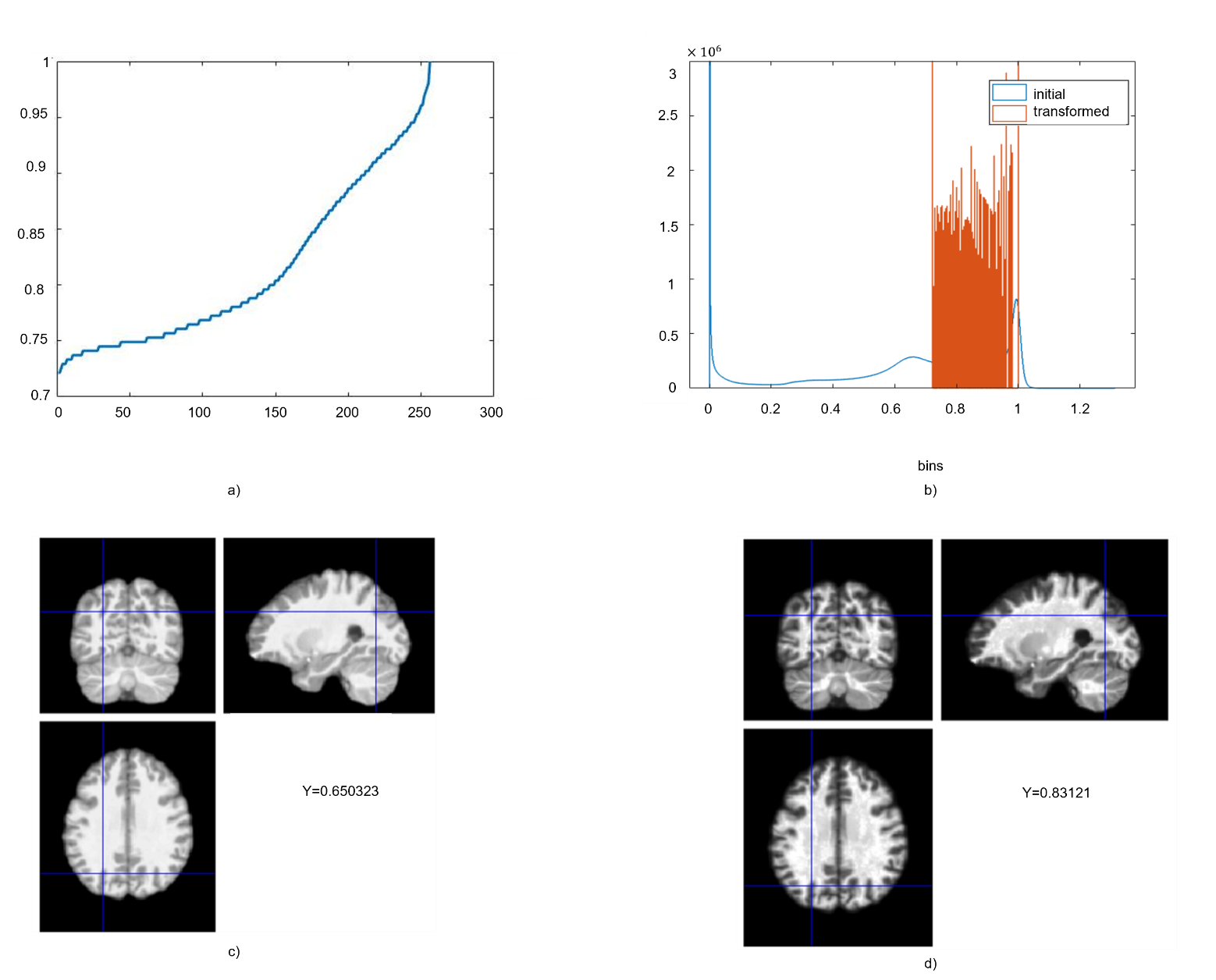


**Figure S2:** Representation of the texture feature maps of energy for a) one ROP+ subject and b) one ROP-, of entropy for c) one CHR-P+ and d) one CHR-P- are presented. Dark colors correspond to lower values of the texture feature, and light colors to higher values of the texture feature.

|  |  |
| --- | --- |
| 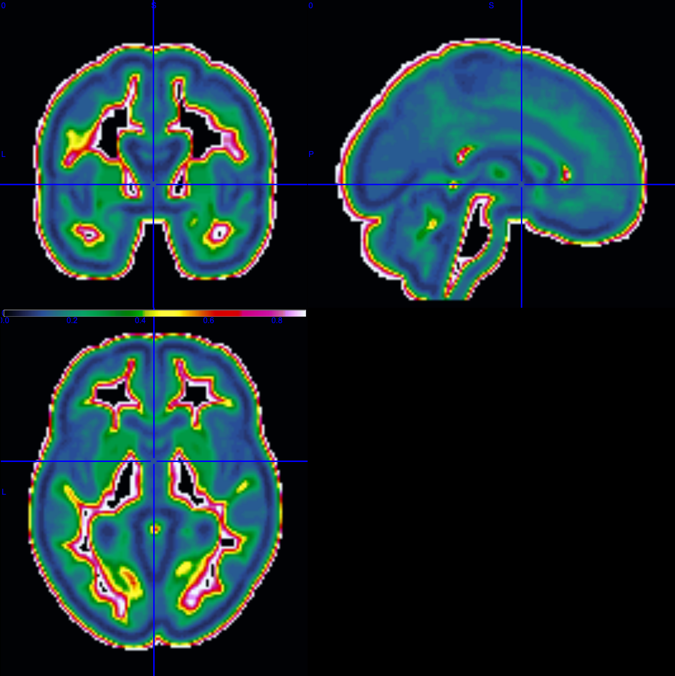   1. Energy in single ROP+ subject   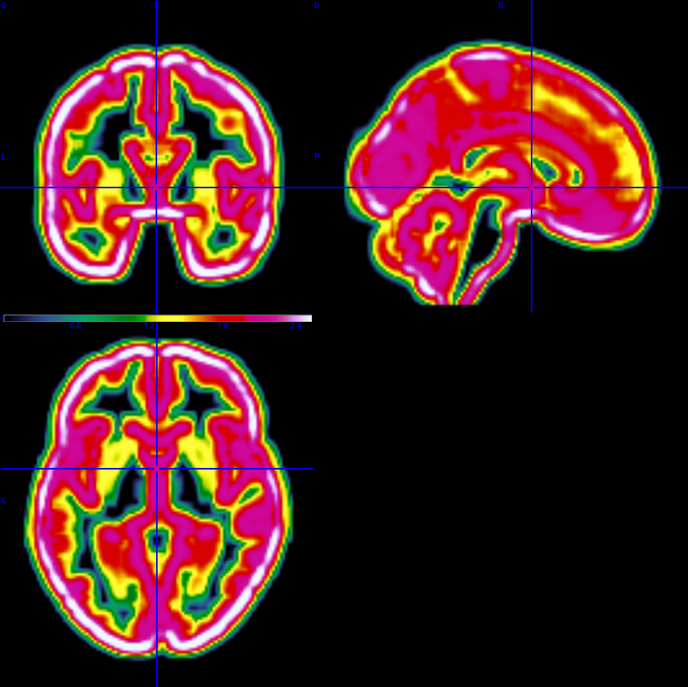  c) Entropy in single CHR-P+ subject | 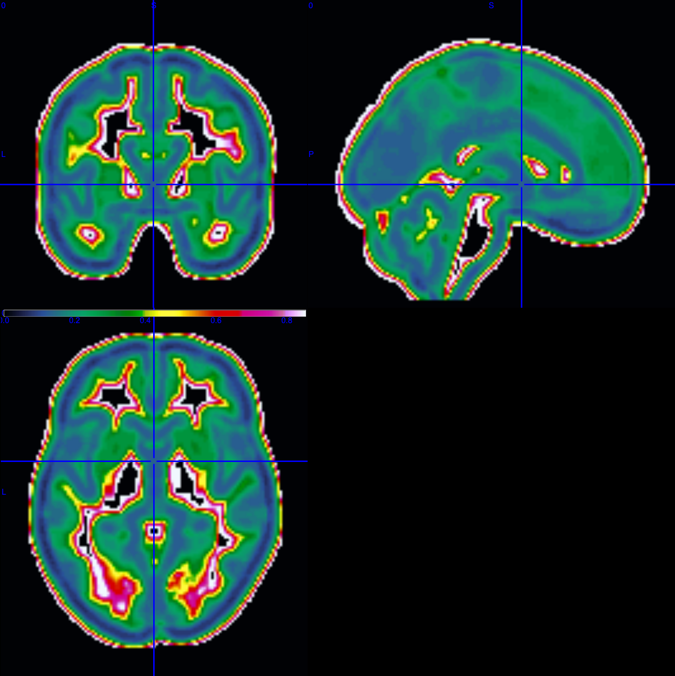  b) Energy in single ROP- subject  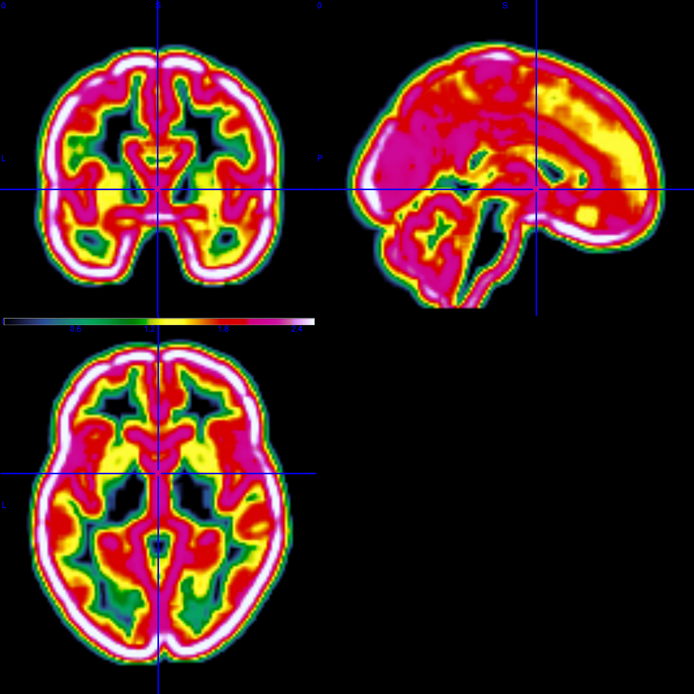  d) Entropy in single CHR-P- subject |

- 1. **Feature extraction and Classification method**

Meaningful comparison of texture feature results between different subjects is possible, if sMRI images of the brain with similar resolution and noise levels are used, a common quantization method and the same number of grey levels in all quantized images are defined^8-11^. We used voxel-by-voxel sliding 3D cube of *7x7x7* dimension as presented in previous papers^12,13^. Where the boundary of the cube touches non-zero brain grey-levels, the algorithm maps the value to the center of the cube. For this reason, a *7x7x7* Gaussian kernel was used to smooth the voxel’s relevance for better localization of the results. The GLCM matrix was normalized by dividing the individual values by the total sum of the values in the matrix. The normalization was performed for each GLCM extracted in each 3D cube independently.

Repeated nested cross-validation (rNCV) was used with 10 outer CV (CV2) permutations, 20 outer CV2 folds, 10 inner CV (CV1) permutations, and 20 inner CV1 folds.

The classifier implemented was a neural network-based classifier implemented in MATLAB (MathWorks Inc., Natick, Massachusetts, USA). The network used the hyperbolic tangent sigmoid transfer function and was batch-trained using the Levenberg-Marquardt training algorithm^14^. L2-regularization was applied to access possible types of uncertainty.

We selected parameters after experimentation; 5 hidden layers (tested 2 to 7), each hidden layer consists of 200 nodes (tested 2 to 500) and 1,000 epochs. Feature selection (two-sample *t*-test) was performed within the inner cross-validation loop by retaining the top 130 ranked features that best discriminated between the two classes. The number of features (130) was chosen empirically to balance model complexity and generalization performance: preliminary analyses indicated that including more features led to overfitting, whereas fewer features reduced classification accuracy. This selection also ensured an appropriate ratio between the number of features and the number of subjects, mitigating the effects of the curse of dimensionality.

**Figure S3**: Preprocessing and feature extraction steps


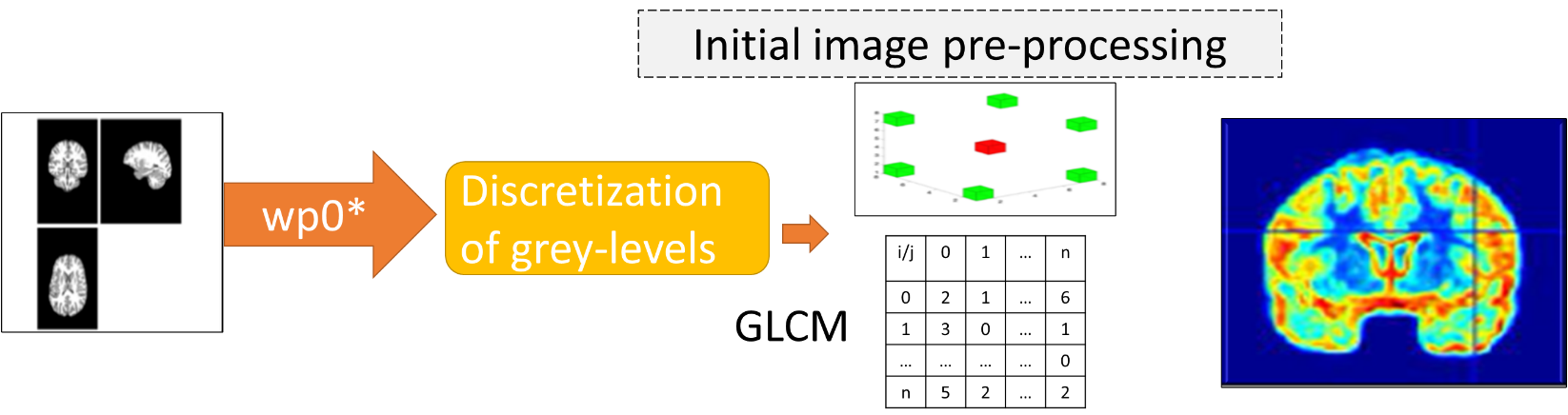


**Figure S4:** Classification schema of the 20x20 repeated 10 times nested cross-validation and explainable artificial intelligence method

Classification framework

Hold-out testing set

Training set split into 20 folds

Validation set to select the winner model

Training set

20 folds

20 folds

|  |  |  |  |
| --- | --- | --- | --- |
| ¨¨¨ | ¨¨¨ | ¨¨¨ | ¨¨¨ |
| ¨¨¨ | ¨¨¨ | ¨¨¨ | ¨¨¨ |
|  |  |  |  |

|  |  |  |  |
| --- | --- | --- | --- |
| ¨¨¨  10 repetitions | ¨¨¨ | ¨¨¨ | ¨¨¨ |
| ¨¨¨ | ¨¨¨ | ¨¨¨ | ¨¨¨ |
|  | Best model selection |  |  |

T-test: 130 ranked voxels selected and validated

T-test: 549 ranked voxels selected and validated

10 repetitions

Explainable Artificial Intelligence


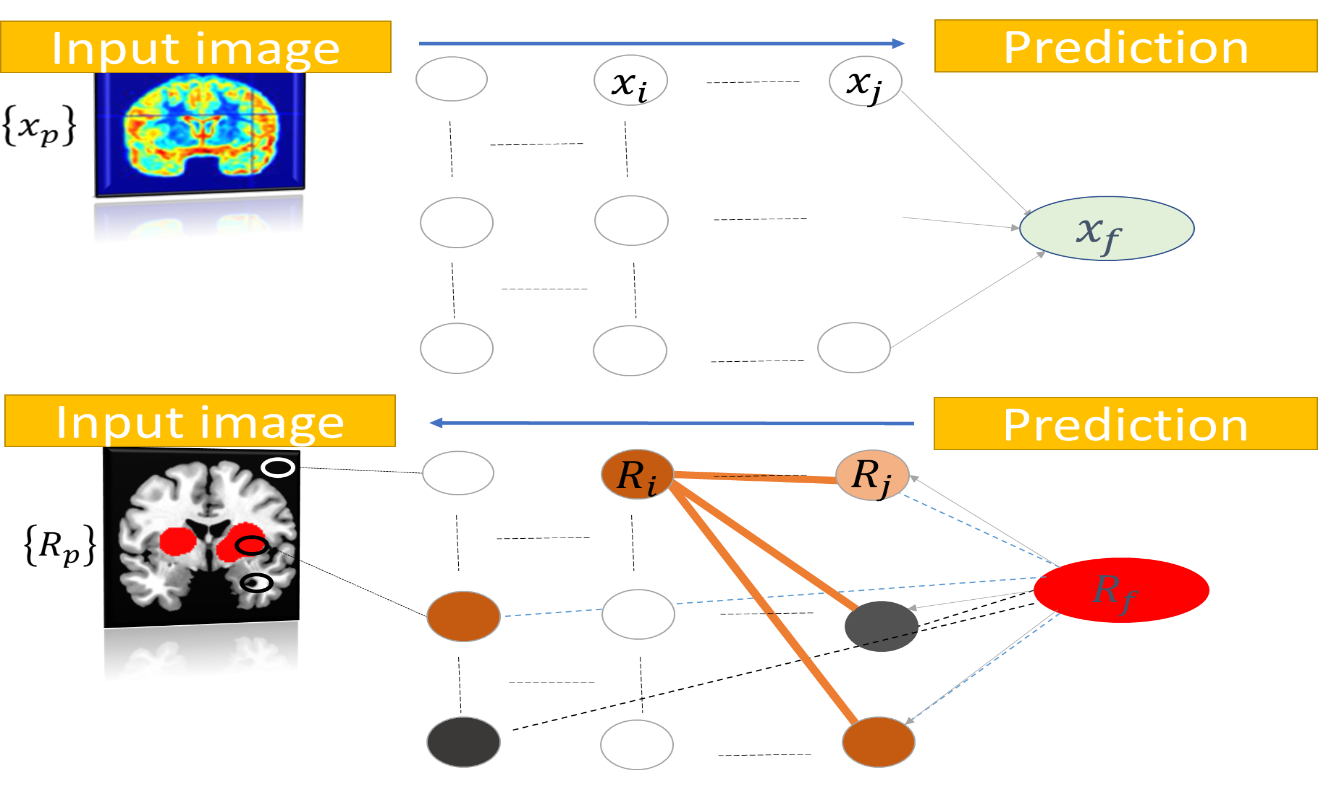


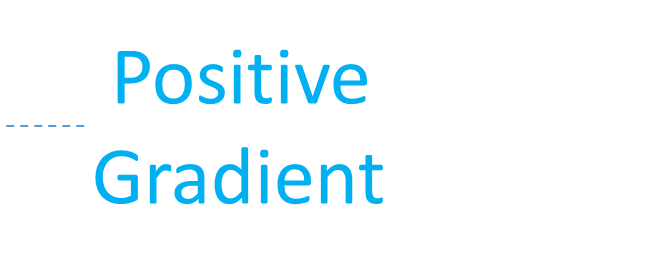

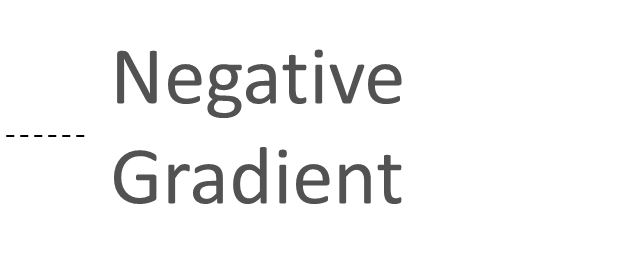

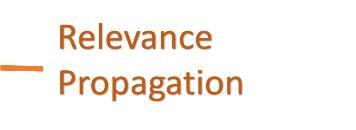


**Table S5.1**: Visualization of the relevance of the corrected classified ROP+ in the external validation sample using AAL-VOIs atlas (https://neurovault.org/images/14257/) on the MRICron.

| Center of mass XYZ 104.31x123.21x102.24 |  | Min | Mean | Max |  | SD |  |  |
| --- | --- | --- | --- | --- | --- | --- | --- | --- |
| VOI nvox(cc)=min/mean/max=SD |  | 0 | 85 | 85 | = | 3 |  |  |
| VOI <>0 nvox(cc)=min/mean/max=SD |  | 0 | 85 | 85 | = | 3 |  |  |
| VOI >0 nvox(cc)=min/mean/max=SD |  | 0 | 85 | 85 | = | 3 |  |  |
| Custom Region Analysis |  |  |  |  |  |  |  |  |
| Index | Name | numVox | numVoxNotZero | fracNotZero | peak | min | mean | meanNotZero |
| 0 |  | 5629168 | 5329 | 0.001 | 0.094118 | 0 | 1.79E-05 | 0.018889 |
| 1 | Precentral_L 2001 | 28174 | 7 | 0 | 0.015686 | 0 | 3.90E-06 | 0.015686 |
| 4 | Frontal_Sup_R 2102 | 32089 | 473 | 0.015 | 0.047059 | 0 | 0.000332 | 0.022518 |
| 6 | Frontal_Sup_Orb_R 2112 | 7859 | 420 | 0.053 | 0.047059 | 0 | 0.001162 | 0.021737 |
| 8 | Frontal_Mid_R 2202 | 40374 | 582 | 0.014 | 0.031373 | 0 | 0.000245 | 0.01698 |
| 10 | Frontal_Mid_Orb_R 2212 | 8057 | 1218 | 0.151 | 0.141176 | 0 | 0.005473 | 0.036202 |
| 11 | Frontal_Inf_Oper_L 2301 | 8271 | 1334 | 0.161 | 0.078431 | 0 | 0.004127 | 0.025587 |
| 12 | Frontal_Inf_Oper_R 2302 | 11174 | 262 | 0.023 | 0.031373 | 0 | 0.000382 | 0.016285 |
| 13 | Frontal_Inf_Tri_L 2311 | 20104 | 23 | 0.001 | 0.015686 | 0 | 1.79E-05 | 0.015686 |
| 14 | Frontal_Inf_Tri_R 2312 | 17132 | 1075 | 0.063 | 0.078431 | 0 | 0.001714 | 0.027316 |
| 16 | Frontal_Inf_Orb_R 2322 | 13747 | 635 | 0.046 | 0.109804 | 0 | 0.001273 | 0.027568 |
| 17 | Rolandic_Oper_L 2331 | 7939 | 47 | 0.006 | 0.015686 | 0 | 9.29E-05 | 0.015686 |
| 24 | Frontal_Sup_Medial_R 2602 | 16979 | 32 | 0.002 | 0.031373 | 0 | 3.33E-05 | 0.017647 |
| 26 | Frontal_Med_Orb_R 2612 | 6870 | 1 | 0 | 0.015686 | 0 | 2.28E-06 | 0.015686 |
| 27 | Rectus_L 2701 | 6864 | 224 | 0.033 | 0.031373 | 0 | 0.00056 | 0.017157 |
| 28 | Rectus_R 2702 | 5930 | 38 | 0.006 | 0.015686 | 0 | 0.000101 | 0.015686 |
| 40 | ParaHippocampal_R 4112 | 9028 | 40 | 0.004 | 0.031373 | 0 | 7.3E-05 | 0.016471 |
| 55 | Fusiform_L 5401 | 18333 | 661 | 0.036 | 0.313726 | 0 | 0.002184 | 0.060562 |
| 57 | Postcentral_L 6001 | 31053 | 1257 | 0.04 | 0.062745 | 0 | 0.000963 | 0.023798 |
| 58 | Postcentral_R 6002 | 30652 | 217 | 0.007 | 0.031373 | 0 | 0.000126 | 0.017855 |
| 61 | Parietal_Inf_L 6201 | 19447 | 76 | 0.004 | 0.031373 | 0 | 6.61E-05 | 0.016925 |
| 64 | SupraMarginal_R 6212 | 15770 | 592 | 0.038 | 0.031373 | 0 | 0.00067 | 0.017859 |
| 67 | Precuneus_L 6301 | 28358 | 580 | 0.02 | 0.031373 | 0 | 0.000347 | 0.016984 |
| 81 | Temporal_Sup_L 8111 | 18307 | 46 | 0.003 | 0.031373 | 0 | 4.03E-05 | 0.016027 |
| 82 | Temporal_Sup_R 8112 | 25258 | 1441 | 0.057 | 0.392157 | 0 | 0.003883 | 0.068068 |
| 84 | Temporal_Pole_Sup_R 8122 | 10654 | 341 | 0.032 | 0.031373 | 0 | 0.000554 | 0.017296 |
| 85 | Temporal_Mid_L 8201 | 39353 | 7045 | 0.179 | 0.658824 | 0 | 0.014742 | 0.08235 |
| 86 | Temporal_Mid_R 8202 | 35484 | 3002 | 0.085 | 0.454902 | 0 | 0.006974 | 0.082429 |
| 88 | Temporal_Pole_Mid_R 8212 | 9470 | 340 | 0.036 | 0.156863 | 0 | 0.00131 | 0.036494 |
| 89 | Temporal_Inf_L 8301 | 25647 | 3278 | 0.128 | 0.54902 | 0 | 0.011898 | 0.093094 |
| 90 | Temporal_Inf_R 8302 | 28468 | 19 | 0.001 | 0.015686 | 0 | 1.05E-05 | 0.015686 |
| 91 | Cerebelum_Crus1_L 9001 | 20667 | 339 | 0.016 | 0.062745 | 0 | 0.000405 | 0.024663 |
| 93 | Cerebelum_Crus2_L 9011 | 15216 | 290 | 0.019 | 0.031373 | 0 | 0.000348 | 0.018283 |
| 99 | Cerebelum_6_L 9041 | 13672 | 64 | 0.005 | 0.047059 | 0 | 8.72E-05 | 0.018627 |

**Table S5.2**: Visualization of the relevance of the corrected classified ROP+ in the external validation sample using the JHU WM tractography atlas on the MRICron.

| Center of mass XYZ 92.37x110.19x81.18 | | Min | Mean | Max |  | SD |  |  |
| --- | --- | --- | --- | --- | --- | --- | --- | --- |
| VOI nvox(cc)=min/mean/max=SD |  | 0 | 0 | 0 | = | 3 |  |  |
| VOI <>0 nvox(cc)=min/mean/max=SD |  | 2147483648 | 0 | -2147483648 | = | 0 |  |  |
| VOI >0 nvox(cc)=min/mean/max=SD |  | 2147483648 | 0 | -2147483648 | = | 0 |  |  |
| Custom Region Analysis | |  |  |  |  |  |  |  |
| Index | Name | numVox | numVoxNotZero | fracNotZero | peak | min | mean | meanNotZero |
| 0 | Unclassified | 7051026 | 30945 | 0.004 | 0.658823568373919 | 0 | 0.000229809428116006 | 0.0523636210273417 |
| 42 | Superior_longitudinal_fasciculus_L | 6605 | 383 | 0.058 | 0.0313725508749485 | 0 | 0.00102358587638931 | 0.0176521794087504 |

**Table S5.3**: Visualization of the relevance of the corrected classified CHR-P+ in the external validation sample using AAL-VOIs atlas (https://neurovault.org/images/14257/) on the MRICron.

| Center of mass XYZ 113x48x14 |  | Min | Mean | Max |  | SD |  |  |
| --- | --- | --- | --- | --- | --- | --- | --- | --- |
| VOI nvox(cc)=min/mean/max=SD |  | 0 | 0 | 0 | = | 1 |  |  |
| VOI <>0 nvox(cc)=min/mean/max=SD |  | 2147483648 | 0 | -2147483648 | = | 0 |  |  |
| VOI >0 nvox(cc)=min/mean/max=SD |  | 2147483648 | 0 | -2147483648 | = | 0 |  |  |
| Custom Region Analysis |  |  |  |  |  |  |  |  |
| Index | Name | numVox | numVoxNotZero | fracNotZero | peak | min | mean | meanNotZero |
| 0 |  | 5629168 | 17103 | 0.003 | 0.800000047311187 | 0 | 0.000241991684253464 | 0.0796475381667369 |
| 1 | Precentral_L 2001 | 28174 | 5 | 0.000 | 0.0313725508749485 | 0 | 3.34058538456895E-6 | 0.0188235305249691 |
| 3 | Frontal_Sup_L 2101 | 28915 | 1062 | 0.037 | 0.062745101749897 | 0 | 0.000927125876626094 | 0.0252427916409072 |
| 5 | Frontal_Sup_Orb_L 2111 | 7654 | 117 | 0.015 | 0.0470588263124228 | 0 | 0.000301265023426798 | 0.0197083973445189 |
| 19 | Supp_Motor_Area_L 2401 | 17282 | 451 | 0.026 | 0.0470588263124228 | 0 | 0.00054187631270525 | 0.0207643158229981 |
| 21 | Olfactory_L 2501 | 2262 | 306 | 0.135 | 0.0784313771873713 | 0 | 0.00308593835971531 | 0.0228117404237779 |
| 25 | Frontal_Med_Orb_L 2611 | 5792 | 4 | 0.001 | 0.0156862754374743 | 0 | 0.000010833063147427 | 0.0156862754374743 |
| 27 | Rectus_L 2701 | 6864 | 574 | 0.084 | 0.10980392806232 | 0 | 0.0036610450540259 | 0.043779465593787 |
| 43 | Calcarine_L 5001 | 18157 | 165 | 0.009 | 0.10980392806232 | 0 | 0.000283367205127034 | 0.0311824142029791 |
| 47 | Lingual_L 5021 | 16932 | 255 | 0.015 | 0.203921580687165 | 0 | 0.000762450075894242 | 0.0506266850393777 |
| 51 | Occipital_Mid_L 5201 | 25989 | 18 | 0.001 | 0.0156862754374743 | 0 | 0.0000108643255944644 | 0.0156862754374743 |
| 53 | Occipital_Inf_L 5301 | 7536 | 50 | 0.007 | 0.062745101749897 | 0 | 0.000147787361473019 | 0.0222745111212134 |
| 71 | Caudate_L 7001 | 7682 | 293 | 0.038 | 0.0784313771873713 | 0 | 0.00109448628410391 | 0.0286957120630928 |
| 73 | Putamen_L 7011 | 7942 | 57 | 0.007 | 0.0313725508749485 | 0 | 0.000126406651724799 | 0.017612660140322 |
| 94 | Cerebelum_Crus2_R 9012 | 17038 | 175 | 0.010 | 0.0784313771873713 | 0 | 0.000208990757383886 | 0.0203473401388952 |
| 102 | Cerebelum_7b_R 9052 | 4230 | 1084 | 0.256 | 0.266666682437062 | 0 | 0.00987530768084963 | 0.0385355641051605 |
| 104 | Cerebelum_8_R 9062 | 18345 | 1619 | 0.088 | 0.345098059624434 | 0 | 0.00479951289346105 | 0.0543836096544428 |
| 105 | Cerebelum_9_L 9071 | 6924 | 16 | 0.002 | 0.0156862754374743 | 0 | 0.0000362478924031756 | 0.0156862754374743 |

**Table S5.4**: Visualization of the relevance of the corrected classified CHR-P+ in the external validation sample using the JHU WM tractography atlas on the MRICron.

| Center of mass XYZ 92.37x110.19x81.18 | | Min | Mean | Max |  | SD |  |  |
| --- | --- | --- | --- | --- | --- | --- | --- | --- |
| VOI nvox(cc)=min/mean/max=SD |  | 0 | 0 | 0 | = | 1 |  |  |
| VOI <>0 nvox(cc)=min/mean/max=SD |  | 2147483648 | 0 | -2147483648 | = | 0 |  |  |
| VOI >0 nvox(cc)=min/mean/max=SD |  | 2147483648 | 0 | -2147483648 | = | 0 |  |  |
| Custom Region Analysis | |  |  |  |  |  |  |  |
| Index | Name | numVox | numVoxNotZero | fracNotZero | peak | min | mean | meanNotZero |
| 0 | Unclassified | 7051026 | 22849 | 0.003 | 0.800000047311187 | 0 | 0.000224810572501518 | 0.0693748169190374 |
| 1 | Middle_cerebellar_peduncle | 15644 | 86 | 0.005 | 0.0313725508749485 | 0 | 0.0000942540201433508 | 0.0171454638502626 |
| 2 | Pontine_crossing_tract_(a_part_of_MCP) | 1500 | 52 | 0.035 | 0.0313725508749485 | 0 | 0.000596078466624022 | 0.017194571152616 |
| 3 | Genu_of_corpus_callosum | 8851 | 12 | 0.001 | 0.0313725508749485 | 0 | 0.0000230393831981884 | 0.0169934650572638 |
| 7 | Corticospinal_tract_R | 1362 | 232 | 0.170 | 0.062745101749897 | 0 | 0.00413463500884967 | 0.0242731589743675 |
| 9 | Medial_lemniscus_R | 690 | 50 | 0.072 | 0.0313725508749485 | 0 | 0.00118215409094009 | 0.0163137264549732 |
| 11 | Inferior_cerebellar_peduncle_R | 968 | 36 | 0.037 | 0.0156862754374743 | 0 | 0.000583373879906067 | 0.0156862754374743 |
| 23 | Anterior_corona_radiata_R | 6849 | 17 | 0.002 | 0.0313725508749485 | 0 | 0.0000435157297871238 | 0.0175317196065889 |
| 33 | External_capsule_R | 5611 | 20 | 0.004 | 0.0470588263124228 | 0 | 0.0000754819883820718 | 0.0211764718405902 |

- 1. **Clustering method**

The affinity propagation (AP) algorithm^15^ uses the concept of information passing between the data, it was selected to cluster the subject’s relevance heatmap for identifying distinct patterns of brain changes related to transdiagnostic psychopathology. The main advantage of the AP algorithm is that the number of clusters is not predefined. The input in the clustering algorithm is a matrix M×N, where N is the number of subjects and M is the relevance of each voxel. The output of the AP algorithm is a scalar for every subject that expresses in which cluster the subject belongs driven only by the values of the brain relevance.

**Table S6:** Clustering analysis in the external validation and T1 samples for the CHR-P+ and ROD+. Association of the clinical variables with the brain relevance heatmaps inside the clusters.

| Cluster | Group at T0 | Symptoms | Outcome Profile after 1-year | Spearman rho-corrected for the prediction of symptoms severity and outcome profiles |
| --- | --- | --- | --- | --- |
| 1 | 6 ROD  7 CHR-P | Oldest and lowest GAF | Improvement of GAF and alogia. High deterioration of PANSS scores |  |
| 2 | 4 ROD  7 CHR-P | Lowest PANSS_distress | High deterioration of PANSS_distress and PANSS_negative. High improvement of avolition. |  |
| 3 | 4 ROD  4 CHR-P | Highest PANSS_disorganization | High deterioration of anhedonia |  |
| 4 | 3 ROD  5 CHR-P | Youngest with high PANSS_distress | High improvement of PANSS_positive and deterioration of blunt affect and alogia. |  |
| 5 | 5 ROD  5 CHR-P | Lowest BDI and anhedonia | High deterioration of BDI and VisDys | Predict the deterioration of PANSS_Positive |
| 6 | 3 ROD  8 CHR-P | Lowest avolition and alogia. Highest VisDys score. | High improvement of anhedonia and distress and VisDys score |  |
| 7 | 5 CHR-P | Highest PANSS and SANS scores | High improvement of PANSS_negative |  |

**Table S7:** Clustering analysis in the external validation and T1 samples for the CHR-P- and ROD-. Association of the clinical variables with the brain relevance heatmaps inside the clusters.

| Cluster | Group at T0 | Symptoms | Outcome Profile after 1-year | Spearman rho-corrected for the prediction of symptoms severity and outcome profiles |  |
| --- | --- | --- | --- | --- | --- |
| 1 | 14 ROD  3 CHR-P | Highest blunting |  |  |  |
| 2 | 21 ROD  9 CHR-P | Younger, highest BDI and lowest PANSS_disorganization, alogia, asociality and anhedonia |  | Predict anhedonia and asociality |  |
| 3 | 6 ROD | Lowest PANSS_negative | High improvement of blunting and deterioration of psychopathology functioning |  |  |
| 4 | 3 ROD  2 CHR-P | Oldest, higher avolition and PANSS_positive | High deterioration of all scores |  |  |
| 5 | 23 ROD  3 CHR-P | Lower BDI | Deterioration of BDI |  |  |
| 6 | 6 ROD  1 CHR-P | Lower PANSS_positive | Improvement of PANSS_negative |  |  |
| 7 | 10 ROD  3 CHR-P |  | High improvement of all PANSS subscores and GAF |  |  |
| 8 | 2 ROD  1 CHR-P | Higher PANSS and SANS scores | High improvement of SANS scores |  |  |
| 9 | 24 ROD  6 CHR-P | Lower PANSS_excitement | Improvement of PANSS_excitement |  |  |
| 10 | 10 ROD  4 CHR-P | Higher GAF | Improvement of PANSS_disorganisation |  |  |

**Figure S5:** The spearman rho correlation results for the p-corrected associations for ROP+/ROD+ at T0 between the rank of a) PANSS_positive, b) PANSS_disorganization, c) PANSS_excitement, d) GAF and the change in follow-up e) PANSS_disorganization at x-axis and the rank of the mean relevance at y-axis.

| **A.**  ****  **ROP+/ROD+**  **PANSS_positive (T0)** | **B.**  ****  **ROP+/ROD+**  **PANSS_disorganisation (T0)** |
| --- | --- |
| **C.**  ****  **ROP+/ROD+**  **PANSS_excitement (T0)** | **D.**  ****  **ROP+/ROD+**  **GAF (T0)** |
| **E.**  ****  **ROP+/ROD+**  **PANSS_disorganization (T1-T0)** | |

**Figure S6:** The average values of the clinical variables at T0 for the combined ROP-/ROD- (blue color) and CHR-P-/ROD- (orange color) that were used for external validation and further associations.

**Figure S7:** The spearman rho correlation results for the p-corrected associations for a) CHR-P+/ROD+ in cluster 5 the change in follow-up for PANSS_positive and in CHR-P-/ROD- b) anhedonia and c) asociality in cluster 2 at x-axis and the brain relevance at y-axis.

| **A.**  ****  **CHR-P+/ROD+ Cluster 5**  **PANSS_positive (T1-T0)** | **B.**  ****  **CHR-P-/ROD- Cluster 2**  **Anhedonia (T1-T0)** |
| --- | --- |
| **C.**  ****  **CHR-P-/ROD- Cluster 2**  **Asociality (T1-T0)** | |

**Figure S8:** The average values of the clinical variables at T0 calculated in each cluster for the CHR-P- subjects belong to the independent validation sample and ROD- subjects. Different colors represent different clusters of brain relevance.

**Figure S9:** The average values of the difference in symptoms in 9 months after the T0 calculated in each cluster, for the CHR-P- subjects belong to the independent validation sample and ROD-. Different colors represent different clusters of brain relevance.

**Table S8**: Spearman rho coefficients and p_values corrected by Bonferonni-Holm for the significant association of whole brain mean relevance heatmaps in CHR-P-/ROD- with the change score from T0 to T1 for anhedonia and asociality from SANS in cluster 2 (* indicates statistical significant p-values).

| **Variable** | **rho** | **t** | **P_corrected** |
| --- | --- | --- | --- |
| SANS_Anhedonia | 0.64 | 3.48 | 0.031* |
| SANS_Asociality | 0.71 | 4.16 | 0.008* |

**Appendix A**

LRP, in its general form assumes that the classifier can be decomposed into several layers of computation. Such layers can be parts of the feature extraction from the image or parts of a classification algorithm run on the calculated features.

For the specific deep learning scheme with 5 hidden layers with size 200, the LRP algorithm is presented:

***Relevance of the 7^th^ Layer***

$${\boldsymbol{R}_{\boldsymbol{j}}}^{\left( \boldsymbol{7} \right)}=f\left( x \right), j=1,2$$

Where the sixth layer is the real-valued prediction output of the classifier $f$ for the two classes $j$.

***Relevance of the 6^th^ Layer between neurons i and j***

For $j=1,2 \mathrm{and} i=1, ..,2$00

$${\boldsymbol{R}_{\boldsymbol{i\leftarrow j}}}^{\left( \boldsymbol{6,7} \right)}\boldsymbol{=}\left\{ \begin{aligned} \frac{z_{ij}}{z_{j}+\varepsilon}{R_{j}}^{\left( 7 \right)}, z_{j}\geq0 \\ \frac{z_{ij}}{z_{j}-\varepsilon}{R_{j}}^{\left( 7 \right)}, z_{j}<0 \end{aligned} \right.,$$

$z_{ij}=x_{i}w_{ij}$,

$$\boldsymbol{z}_{\boldsymbol{j}}\boldsymbol{=}\sum_{i=1}^{2} z_{ij}+b_{j}$$

Where $x_{i}$ is the output of the fifth hidden layer using the *tansig* transfer function on the net input, $w_{ij}$ are the weights and $b_{j}$ the biases of the neurons connect the fourth and third layer. $\varepsilon$ is *0.001* just to avoid the division with zero. So, the voxel-wise relevance in the third hidden layer is calculated as:

$${\boldsymbol{R}_{\boldsymbol{i}}}^{\boldsymbol{(6)}}\boldsymbol{=}\sum_{j=1}^{2} {R_{i\leftarrow j}}^{\left( 6,7 \right)}$$

***Relevance of the 5^th^ Layer between neurons i and k***

For $i=1,..,200 \mathrm{and} k=1,\ldots,200$

$${\boldsymbol{R}_{\boldsymbol{k\leftarrow i}}}^{\left( \boldsymbol{5,6} \right)}=\left\{ \begin{aligned} \frac{z_{ki}}{z_{i}+\varepsilon}{R_{i}}^{\left( 6 \right)}, z_{i}\geq0 \\ \frac{z_{ki}}{z_{i}-\varepsilon}{R_{i}}^{\left( 6 \right)}, z_{i}<0 \end{aligned} \right.,$$

$z_{ki}=x_{k}w_{ki}$,

$$\boldsymbol{z}_{\boldsymbol{i}}=\sum_{k=1}^{200} z_{ki}+b_{i}$$

Where $x_{k}$ is the output of the fourth hidden layer using the *tansig* transfer function on the net input, $w_{ki}$ are the weights and $b_{i}$ the biases of the neurons connect the second and third layer. $\varepsilon$ is *0.001* just to avoid the division with zero. So, the voxel-wise relevance in the second hidden layer is calculated as:

$${\boldsymbol{R}_{\boldsymbol{k}}}^{\left( \boldsymbol{5} \right)}=\sum_{i=1}^{200} {R_{k\leftarrow i}}^{\left( 5,6 \right)}$$

***Relevance of the 4^th^ Layer between neurons i and k***

For $i=1,..,200 \mathrm{and} k=1,\ldots,200$

$${\boldsymbol{R}_{\boldsymbol{k\leftarrow i}}}^{\left( \boldsymbol{4,5} \right)}=\left\{ \begin{aligned} \frac{z_{ki}}{z_{i}+\varepsilon}{R_{i}}^{\left( 5 \right)}, z_{i}\geq0 \\ \frac{z_{ki}}{z_{i}-\varepsilon}{R_{i}}^{\left( 5 \right)}, z_{i}<0 \end{aligned} \right.,$$

$z_{ki}=x_{k}w_{ki}$,

$$\boldsymbol{z}_{\boldsymbol{i}}=\sum_{k=1}^{200} z_{ki}+b_{i}$$

Where $x_{k}$ is the output of the third hidden layer using the *tansig* transfer function on the net input, $w_{ki}$ are the weights and $b_{i}$ the biases of the neurons connect the second and third layer. $\varepsilon$ is *0.001* just to avoid the division with zero. So, the voxel-wise relevance in the second hidden layer is calculated as:

$${\boldsymbol{R}_{\boldsymbol{k}}}^{\left( \boldsymbol{4} \right)}=\sum_{i=1}^{200} {R_{k\leftarrow i}}^{\left( 4,5 \right)}$$

***Relevance of the 3^rd^ Layer between neurons i and k***

For $i=1,..,200 \mathrm{and} k=1,\ldots,200$

$${\boldsymbol{R}_{\boldsymbol{k\leftarrow i}}}^{\left( \boldsymbol{3,4} \right)}=\left\{ \begin{aligned} \frac{z_{ki}}{z_{i}+\varepsilon}{R_{i}}^{\left( 4 \right)}, z_{i}\geq0 \\ \frac{z_{ki}}{z_{i}-\varepsilon}{R_{i}}^{\left( 4 \right)}, z_{i}<0 \end{aligned} \right.,$$

$z_{ki}=x_{k}w_{ki}$,

$$\boldsymbol{z}_{\boldsymbol{i}}=\sum_{k=1}^{200} z_{ki}+b_{i}$$

Where $x_{k}$ is the output of the second hidden layer using the *tansig* transfer function on the net input, $w_{ki}$ are the weights and $b_{i}$ the biases of the neurons connect the second and third layer. $\varepsilon$ is *0.001* just to avoid the division with zero. So, the voxel-wise relevance in the second hidden layer is calculated as:

$${\boldsymbol{R}_{\boldsymbol{k}}}^{\left( \boldsymbol{3} \right)}=\sum_{i=1}^{200} {R_{k\leftarrow i}}^{\left( 3,4 \right)}$$

***Relevance of the 2^nd^ Layer between neurons k and l***

For $k=1,..,200 \mathrm{and} l=1,\ldots,200$

$${\boldsymbol{R}_{\boldsymbol{l\leftarrow k}}}^{\left( \boldsymbol{2,3} \right)}=\left\{ \begin{aligned} \frac{z_{lk}}{z_{k}+\varepsilon}{R_{k}}^{\left( 3 \right)}, z_{k}\geq0 \\ \frac{z_{lk}}{z_{k}-\varepsilon}{R_{k}}^{\left( 3 \right)}, z_{k}<0 \end{aligned} \right.,$$

$z_{lk}=x_{l}w_{lk}$,

$$\boldsymbol{z}_{\boldsymbol{k}}=\sum_{l=1}^{200} z_{lk}+b_{k}$$

Where $x_{l}$ is the output of the first hidden layer using the *tansig* transfer function on the net input, $w_{lk}$ are the weights and $b_{k}$ the biases of the neurons connect the second and third layer. $\varepsilon$ is *0.001* just to avoid the division with zero. So, the voxel-wise relevance in the first hidden layer is calculated as:

$${\boldsymbol{R}_{\boldsymbol{l}}}^{\left( \boldsymbol{2} \right)}=\sum_{k=1}^{2} {R_{l\leftarrow k}}^{\left( 2,3 \right)}$$

***Relevance of the 1^st^ Layer between input voxels and neurons l***

For $d=1,\ldots,549$ voxels:

$${\boldsymbol{R}_{\boldsymbol{d\leftarrow l}}}^{\left( \boldsymbol{1,2} \right)}=\left\{ \begin{aligned} \frac{z_{dl}}{z_{l}+\varepsilon}{R_{l}}^{\left( 2 \right)}, z_{l}\geq0 \\ \frac{z_{dl}}{z_{l}-\varepsilon}{R_{l}}^{\left( 2 \right)}, z_{l}<0 \end{aligned} \right.,$$

$z_{dl}=x_{d}w_{dl}$,

$$\boldsymbol{z}_{\boldsymbol{l}}=\sum_{d=1}^{549} z_{dl}+b_{l}$$

Where $x_{d}$ is the input registered texture feature map based image, $w_{dl}$ are the weights and $b_{l}$ the biases of the neurons connect the input and second layer. So, the voxel-wise relevance in the input layer is calculated as:

$${\boldsymbol{R}_{\boldsymbol{d}}}^{\left( \boldsymbol{1} \right)}=\sum_{l=1}^{200} {R_{d\leftarrow l}}^{\left( 1,2 \right)}$$

**References**

1 Regier, D. A., Kuhl, E. A. & Kupfer, D. J. The DSM-5: Classification and criteria changes. *World Psychiatry* **12**, 92-98 (2013). <https://doi.org:https://doi.org/10.1002/wps.20050>

2 Miller, T. J. *et al.* Prodromal Assessment With the Structured Interview for Prodromal Syndromes and the Scale of Prodromal Symptoms: Predictive Validity, Interrater Reliability, and Training to Reliability. *Schizophrenia Bulletin* **29**, 703-715 (2003). <https://doi.org:10.1093/oxfordjournals.schbul.a007040>

3 Lencer, R. *et al.* Saccadic suppression in schizophrenia. *Scientific Reports* **11**, 13133 (2021). <https://doi.org:10.1038/s41598-021-92531-2>

4 Wallwork, R. S., Fortgang, R., Hashimoto, R., Weinberger, D. R. & Dickinson, D. Searching for a consensus five-factor model of the Positive and Negative Syndrome Scale for schizophrenia. *Schizophrenia Research* **137**, 246-250 (2012). <https://doi.org:https://doi.org/10.1016/j.schres.2012.01.031>

5 Lalousis, P. A. *et al.* Neurobiologically Based Stratification of Recent Onset Depression and Psychosis: Identification of Two Distinct Transdiagnostic Phenotypes. *Biological Psychiatry* (2022). <https://doi.org:https://doi.org/10.1016/j.biopsych.2022.03.021>

6 Manjón, J. V. *et al.* Robust MRI brain tissue parameter estimation by multistage outlier rejection. *Magnetic Resonance in Medicine* **59**, 866-873 (2008). <https://doi.org:https://doi.org/10.1002/mrm.21521>

7 Rajapakse, J. C., Giedd, J. N. & Rapoport, J. L. Statistical approach to segmentation of single-channel cerebral MR images. *IEEE Transactions on Medical Imaging* **16**, 176-186 (1997). <https://doi.org:10.1109/42.563663>

8 Brynolfsson, P. *et al.* Haralick texture features from apparent diffusion coefficient (ADC) MRI images depend on imaging and pre-processing parameters. *Scientific Reports* **7**, 4041 (2017). <https://doi.org:10.1038/s41598-017-04151-4>

9 Traverso, A., Wee, L., Dekker, A. & Gillies, R. Repeatability and Reproducibility of Radiomic Features: A Systematic Review. *International Journal of Radiation Oncology*Biology*Physics* **102**, 1143-1158 (2018). <https://doi.org:https://doi.org/10.1016/j.ijrobp.2018.05.053>

10 Buch, K., Kuno, H., Qureshi, M. M., Li, B. & Sakai, O. Quantitative variations in texture analysis features dependent on MRI scanning parameters: A phantom model. *Journal of Applied Clinical Medical Physics* **19**, 253-264 (2018). <https://doi.org:https://doi.org/10.1002/acm2.12482>

11 Rizzo, S. *et al.* Radiomics: the facts and the challenges of image analysis. *European Radiology Experimental* **2**, 36 (2018). <https://doi.org:10.1186/s41747-018-0068-z>

12 Korda, A. I. *et al.* Identification of voxel-based texture abnormalities as new biomarkers for schizophrenia and major depressive patients using layer-wise relevance propagation on deep learning decisions. *Psychiatry Research: Neuroimaging* **313**, 111303 (2021). <https://doi.org:https://doi.org/10.1016/j.pscychresns.2021.111303>

13 Korda Alexandra I *et al.* Identification of texture MRI brain abnormalities on first-episode psychosis and clinical high-risk subjects using explainable artificial intelligence. *Translational Psychiatry* **12**, 481 (2022). <https://doi.org:10.1038/s41398-022-02242-z>

14 Lourakis, M. A Brief Description of the Levenberg-Marquardt Algorithm Implemened by levmar. *A Brief Description of the Levenberg-Marquardt Algorithm Implemented by Levmar* **4** (2005).

15 Frey Brendan, J. & Dueck, D. Clustering by Passing Messages Between Data Points. *Science* **315**, 972-976 (2007). <https://doi.org:10.1126/science.1136800>
